# Bryostatin-1 enhances the proliferation and functionality of exhausted CD8+ T cells by upregulating MAP Kinase 11

**DOI:** 10.3389/fimmu.2024.1509874

**Published:** 2025-01-14

**Authors:** Ling Li, Manzhi Zhao, Marjan van Meurs, Inge Brouwers-Haspels, Renske J. H. den Dekker, Merel E. P. Wilmsen, Dwin G. B. Grashof, Harmen J. G. van de Werken, Shringar Rao, Casper Rokx, Yvonne M. Mueller, Peter D. Katsikis

**Affiliations:** ^1^ Department of Immunology, Erasmus University Medical Center, Rotterdam, Netherlands; ^2^ Department of Biochemistry, Erasmus University Medical Center, Rotterdam, Netherlands; ^3^ Department of Internal Medicine, and Department of Medical Microbiology and Infectious Diseases, Erasmus University Medical Center, Rotterdam, Netherlands

**Keywords:** bryostatin-1, exhausted CD8+ T cells, HIV, MAP kinase 11, IFN-γ production

## Abstract

**Introduction:**

Bryostatin-1, a potent agonist of the protein kinase C, has been studied for HIV and cancer therapies. In HIV research, it has shown anti-HIV effects during acute infection and reactivation of latent HIV in chronic infection. As effective CD8+ T cell responses are essential for eliminating reactivated virus and achieving a cure, it is important to investigate how bryostatin-1 affects HIV-specific CD8+ T cells. HIV-specific CD8+ T cells often become exhausted, showing reduced proliferative potential and impaired cytokine production, a dysfunction also observed in cancer. Therefore, we further investigated how bryostatin-1 directly impacts exhausted CD8+ T cells.

**Methods:**

PBMCs from people with HIV (PWH) were treated with bryostatin-1 and tracked with proliferation dye for cell expansion. One day 6, HIV-specific CD8+ T cells were detected by tetramers staining and examined by flow cytometry. By utilizing an established *in vitro* murine T cell exhaustion system, changes in inhibitory receptors, transcription factors, cytokine production and killing capacity of bryostatin-1 treated exhausted CD8+ T cells were determined by flow cytometry. RNA-seq analysis was performed to study transcriptional changes in these cells.

**Results:**

We found that bryostatin-1 improved the expansion and decreased PD-1 expression of HIV-specific CD8+ T cells. Bryostatin-1 enhanced the functionality and proliferation while decreasing inhibitory receptor expression of *in vitro* generated exhausted CD8+ T cells. Bryostatin-1 upregulated TCF-1 and decreased TOX expression. These changes were confirmed through RNA-seq analysis. RNA-seq revealed that mitogen-activated protein kinases (MAPK) 11 was significantly downregulated in exhausted CD8+ T cells, however, it greatly upregulated after bryostatin-1 treatment. Inhibition of MAPK11 in bryostatin-1-treated cells blocked the increased proliferation and IFN-γ production induced by bryostatin-1, but did not affect other bryostatin-1 induced effects, such as the reduction of inhibitory receptors.

**Discussion:**

Our data demonstrate that bryostatin-1 induces a MAPK 11-dependent improvement in the proliferative and functional capacity of exhausted T cells. This study provides a rationale for bryostatin-1's potential to help eradicate the HIV reservoir during treatment, and it may also contribute to cancer immunotherapy by functionally improving exhausted CD8+ T cells.

## 1 Introduction

CD8+ T cells can become exhausted when they are persistently exposed to antigen, as in chronic infections and cancer (1–3). Exhausted CD8+ T cells are characterized by sustained expression of inhibitory receptors, diminished effector function and a distinct transcriptional state different from effector or memory cells (4). The dysfunctional state of CD8+ T cells compromises their ability to effectively control infections (*e.g.* HIV infection) and cancer. Therefore, reinvigorating exhausted CD8+ T cells to enhance T cell immunity holds the potential to restore their ability to control disease progression (5–7).

Bryostatin-1 is a bioactive macrocyclic lactone derived from marine organisms, exhibiting a wide range of pharmacological activities (8). Bryostatin-1 acts as a potent agonist of the protein kinase C (PKC) family. Upon binding to PKC, it rapidly triggers the activation of PKC, leading to its auto-phosphorylation and translocation from the cytosol to the plasma membrane (9, 10). However, during prolonged exposure, it can also mediate PKC downregulation through proteasomal degradation (11). Additionally, bryostatin-1 interacts with other targets such as protein kinase D and acts as a Toll-like receptor 4 (TLR-4) ligand (12, 13). Bryostatin-1 has been investigated in numerous clinical trials for cancer therapy due to its antitumor activity and immunomodulatory effects (14, 15). It induces cell differentiation of malignant hematopoietic cells and inhibits cancer cell proliferation, directly suppressing tumor growth in preclinical experiments (16–18). Furthermore, bryostatin-1 enhances anti-tumor immunity by stimulating cytotoxic CD8+ T cells by inducing T cell proliferation and IL-2 receptor upregulation, which is dependent on PKC activation (14, 19–21). Tumor-specific T cells, expanded *ex vivo* by bryostatin-1 and ionomycin (B/I) and subsequently adoptively transferred into tumor-bearing mice, mediate tumor regression (22). B/I preferentially expands CD62L^low^ tumor-sensitized CD8+ T cells during *ex vivo* stimulation and these can play an essential role in tumor control (23). Although bryostatin-1 monotherapy for human cancer treatment has not shown significant clinical benefits in phase II clinical trials (24–28), there may still be potential for its efficacy when used in combination with other agents (29).

Bryostatin-1 can also robustly reactivate latent human immunodeficiency virus (HIV), the main obstacle to an HIV cure, by activating NF-κB and PKC (30, 31). Reversing HIV latency through latency reversal agents (LRAs), like bryostatin-1, is expected to aid in eradicating HIV infected cells, contributing to the “shock and kill” strategy (32). This strategy involves “shocking” latent HIV out of its dormant state, making the infected cells visible to the host immune system, where they can be targeted and killed by CD8+ T cells, potentially bringing us closer to a cure (33). Bryostatin-1 has also been investigated as an HIV inhibitor and demonstrates anti-HIV activity against X4- and R5-tropic strains in a receptor-independent manner (31). Bryostatin-1 transiently downregulates CD4 receptors and CXCR4 co-receptors on Jurkat cells, partially contributing to its anti-HIV effect (31). Additionally, bryostatin-1 reduces CD4 and CCR5 receptor expression in human macrophages, leading to decreased HIV infection (34). However, in contrast to its HIV-reactivating effect in CD4+ T cells, bryostatin-1 does not increase viral production in chronically HIV infected macrophages (34). Bryostatin-1 induces latent HIV reactivation while suppressing HIV infectivity in T cells or macrophages at low nanomolar concentrations (31, 34). In a previous study, we demonstrated that bryostatin-1 shows no significant cytotoxicity on either unactivated or activated CD8+ T cells, as well as on other immune subpopulations such as B cells and NK cells (35). Importantly, bryostatin-1 downregulated inhibitory receptor PD-1 expression on activated CD8+ T cells from people with HIV (PWH) without impeding their activation, suggesting that byrostatin-1 may modulate the function of those cells (35). Since HIV-specific CD8+ T cells become functionally exhausted during chronic HIV infection with elevated levels of immune checkpoints such as PD-1 and diminished proliferative capacity (36–38), we decided to investigate how bryostatin-1 impacts the dysfunctional state of HIV-specific CD8+ T cells.

In this study, we found that bryostatin-1 expanded HIV-specific CD8+ T cells and decreased the expression of PD-1 on those cells during long-term culture in the presence of CD28 costimulation. To further investigate whether bryostatin-1 reduces CD8+ T cell exhaustion, we utilized an *in vitro* murine T cell exhaustion assay (39). Our results show that bryostatin-1 reduces expression of multiple inhibitory receptors (PD-1, TIGIT, CD160 and LAG-3) on exhausted CD8+ T cells, and increases cell expansion and functionality by improving cytokine production and cytotoxicity. Inhibition of mitogen-activated protein kinases (MAPK) 11 can block the bryostatin-1-mediated improved proliferation and IFN-γ production of exhausted cells. Our findings reveal that bryostatin-1 reinvigorates exhausted CD8+ T cells and expands HIV-specific CD8+ T cells, suggesting that byrostatin-1 may be beneficial in the treatment of HIV infection and may also improve immunotherapy in cancers.

## 2 Results

### 2.1 Bryostatin-1 preferentially expands HIV-specific CD8+ T cells

**Figure 1:**
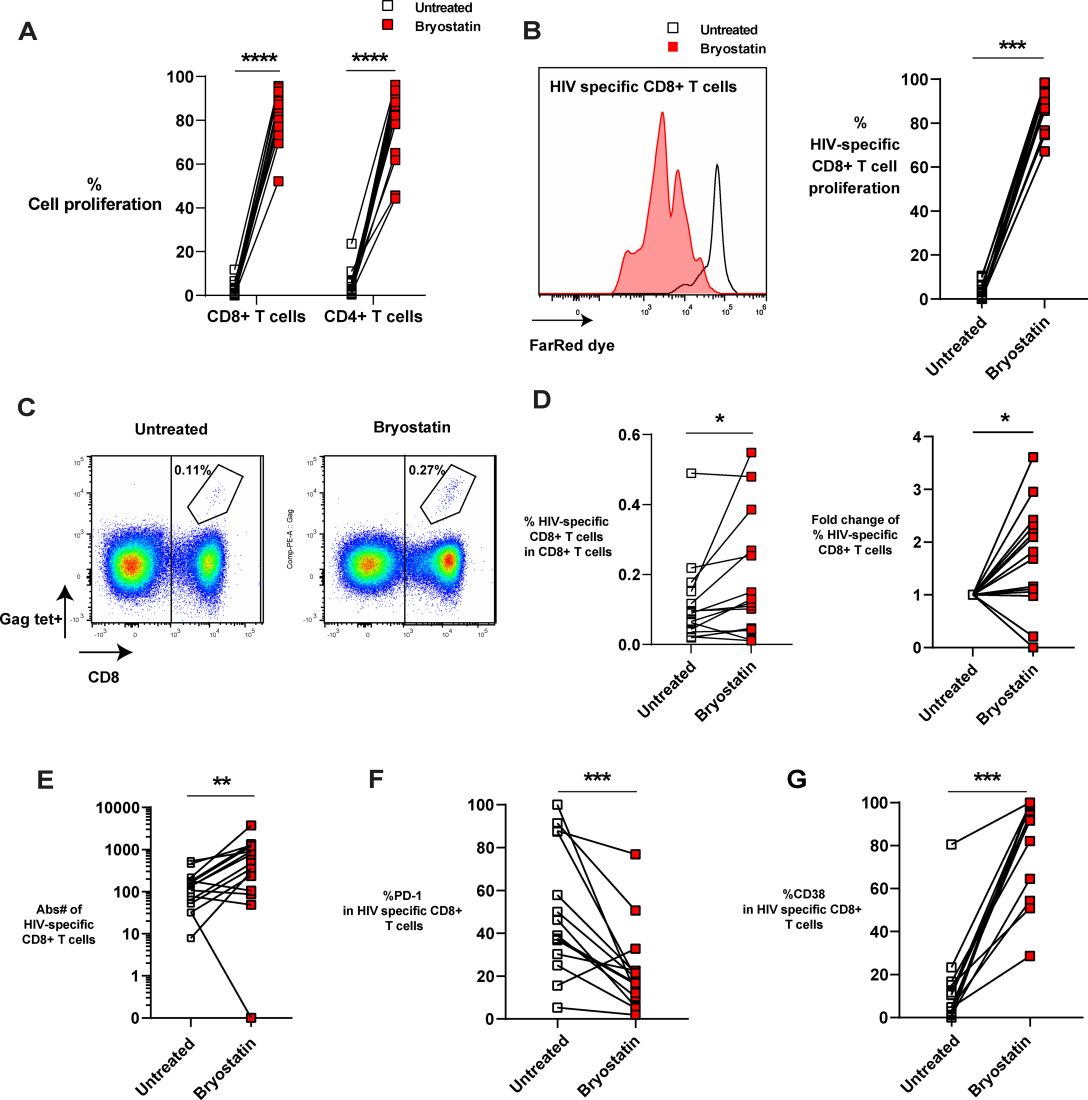
Bryostatin-1 expands HIV- specific CD8+ T cells in the presence of CD28 costimulation. PBMCs from PWH were either untreated or treated with bryostatin-1 for 6 days. On day 0, cells were labeled with Far Red proliferation dye to track their proliferation. On day 6, cells were harvested and analyzed by flow cytometry. **(A)** The proliferation of both CD8+ T cells and CD4+ T cells is shown. % Cell proliferation represents the percentage of CD4+ or CD8+ T cells which underwent at least one division with or without bryostatin-1 treatment during the six days culture. **(B)** Representative histogram (left) and pooled data (right) depicting the proliferation of HIV-specific CD8+ T cells on day 6 with or without bryostatin-1 treatment. **(C)** Representative FACS plots of the frequency of HIV-specific CD8+ T cells (Gag-Specific HLA-A*02:01 Tetramer+). **(D)** The percentage of HIV-specific CD8+ T cells of total CD8+ T cells treated with DMSO or bryostatin-1 is depicted (left). The fold change in the percentage of HIV-specific CD8+ T cells among total CD8+ T cells after treatment with bryostatin-1 compared to the untreated condition is shown (right). **(E)** Absolute number of HIV-specific T cells is shown. **(F)** The percentage of PD-1 expression in HIV-specific CD8+ T cells is shown. **(G)** Pooled data showing the frequency of CD38 in HIV-specific CD8+ T cells in culture. Each symbol represents one HIV+ donor. In **(A, D, E)** n=15; In **(B, F, G)** n=14 (due to one donor having no HIV-specific CD8+ T cells on day 6). Wilcoxon matched-pairs signed rank test was performed to test for statistical significance. * for p < 0.05, ** for p < 0.01, *** for p < 0.001 and **** for p < 0.001.

Our previous study has shown that bryostatin-1 significantly reduced inhibitory receptor PD-1 expression on activated CD8+ T cells from PWH at a concentration of 10 nM in a three days culture (35). To investigate how bryostatin-1 affects HIV-specific CD8+ T cells, we treated HLA-A*02:01 PBMCs from PWH with 10 nM bryostatin-1 or DMSO in the presence of anti-CD28 antibodies for 6 days. We combined bryostatin-1 with costimulation as it was shown that byrostatin-1 alone exhibited a marginal proliferative effect, but together with CD28 costimulation bryostatin-1 promoted T cell proliferation (40). We show here that bryostatin-1 at the concentration of 10 nM together with CD28 costimulation induced a vigorous proliferative response in CD4+ and CD8+ T cells, and this enhanced proliferation of T cells by bryostatin-1 was also observed at lower concentrations (**Figure 1A**; **Supplementary Figure S1A, B**). Although the increase in cell proliferation was consistently observed, bryostatin-1 did not significantly impact cell survival in these patients (**Supplementary Figure S1C**). We measured HIV-specific CD8+ T cells by Gag SL9 or Pol IV9 HLA-A*02:01 tetramers staining. We found that bryostatin-1 with anti-CD28 antibody proliferated HIV-specific CD8+ T cells in fourteen out of fifteen patients and did not significantly change cell viability of HIV-specific CD8+ T cells (**Figure 1B**; **Supplementary Figure S1D**). Importantly, bryostatin-1 significantly increased the frequency of HIV-specific CD8+ T cells by an average of 1.65-fold (**Figures 1C, D**), as well as the absolute number of these cells (**Figure 1E**), suggesting that bryostatin-1 preferentially expanded HIV-specific CD8+ T cells compared to the total CD8+ T cell population.

The inhibitory receptor PD-1, which is associated with T cell exhaustion, is upregulated on HIV-specific T cells (37, 41). We found that *in vitro* treatment with bryostatin-1 downregulated PD-1 expression level on HIV-specific T cells while increasing the chronic activation marker CD38 (**Figures 1F, G**). Taken together, our data show that bryostatin-1 combined with CD28 costimulation preferentially expands HIV-specific CD8+ T cells and reduces exhaustion-associated features of HIV-specific T cells as it increases proliferation and decreases PD-1 expression, without blocking the activation of these cells.

### 2.2 Bryostatin-1 reduces exhaustion-related phenotypes in exhausted CD8+ T cells *in vitro*

**Figure 2:**
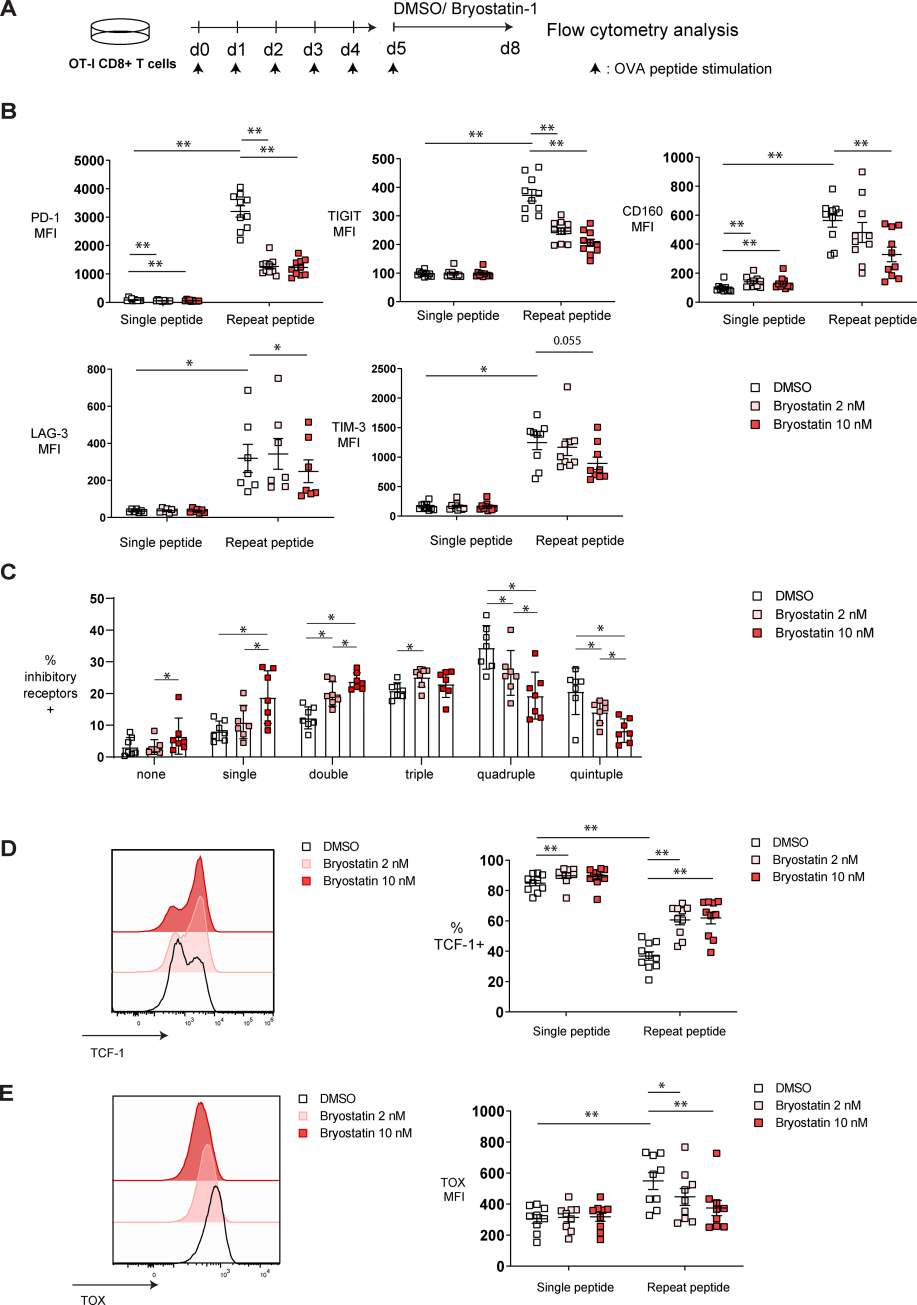
Bryostatin-1 decreases exhaustion-associated markers in *in vitro* exhausted T cells. Exhausted and non-exhausted OT-I T cells were generated *in vitro* by either repeat or single OVA _(257-264)_ peptide stimulation. From day 5, cells were either treated with 2 nM bryostatin-1, 10 nM bryostatin-1 or DMSO for additional 3 days. Exhaustion-associated markers of those cells were determined by flow cytometry on day 8. **(A)** Scheme of testing the effect of bryostatin-1 in *in vitro* murine exhausted T cells. **(B)** Pooled data showing the median fluorescence intensity (MFI) of inhibitory receptors PD-1, TIGIT, CD160, LAG-3 and TIM-3, on DMSO or bryostatin-1 treated cells. **(C)** Bar chart depicting frequency of DMSO or byostatin-1 treated repeat peptide stimulated cells expressing either one, two, three, four or five of the inhibitory receptors including PD-1, TIGIT, CD160, LAG-3 and TIM-3. **(D)** Representative histograms (left) depicting the expression of TCF-1 in exhausted T cells after bryostatin-1 treatment. Pooled data (right) showing the changes in the frequencies of TCF-1+ CD8+ T cells after bryostatin-1 treatment. **(E)** Representative histograms (left) depicting the expression of TOX in exhausted T cells that were treated either with bryostatin-1 or DMSO. Pooled data (right) showing the change of the TOX expression in CD8+ T cells after bryostatin-1 treatment. Each symbol represents one animal (n=7-10), 7 independent experiments were performed. Lines depict mean ± SE. Between groups, Wilcoxon matched-pairs signed rank test was performed as statistical tests. In figure **(B, D, E)** statistical comparisons were made between single-peptide DMSO control and repeat-peptide DMSO control, as well as between single- or repeat-peptide conditions. * for p < 0.05, ** for p < 0.01.

Since bryostatin-1 downregulated PD-1 expression on HIV-specific CD8+ T cells, and PD-1 expression is known to be related to the exhaustion state of these cells (36, 42), we hypothesized that bryostatin-1 may modulate CD8+ T cell exhaustion. To investigate whether bryostatin-1 directly acts on exhausted T cells, we utilized an *in vitro* exhaustion system in which murine CD8+ T cells were induced to become exhausted (39). In this system, OT-I cells undergo exhaustion when repeatedly stimulated with cognate OVA _(257–264)_ peptide for 5 consecutive days. As controls for the exhausted CD8+ T cells, cells were stimulated only once with OVA _(257-264)_ peptide during the first two days, then washed and left unstimulated for the subsequent three days to serve as the non-exhausted controls. From day 5 to day 8, both exhausted and non-exhausted OT-I cells were either treated with 2 or 10 nM bryostatin-1 or DMSO. The exhaustion phenotypic changes of OT-I cells were examined by flow cytometry analysis on day 8 (**Figure 2A**; **Supplementary Figure S2A**). As expected, the inhibitory receptors PD-1, TIGIT, CD160, LAG-3, and TIM-3 were substantially upregulated in cells that were repeatedly stimulated with peptide compared to cells that received only a single time peptide stimulation (**Figure 2B**). This indicates the successful induction of the exhaustion phenotype in cells repeatedly stimulated with peptide in our assay. When cells were treated with 10 nM bryostatin-1, the expression of PD-1, TIGIT, CD160, and LAG-3 was significantly decreased in exhausted repeat peptide stimulated cells compared to cells treated with DMSO control. PD-1 and TIGIT were downregulated also at the lower 2 nM dose of bryostatin-1 (**Figure 2B**; **Supplementary Figure S2B**). Bryostatin-1 treatment decreased the frequency of exhausted T cells that co-express multiple inhibitory receptors. Exhausted T cells co-expressing four and five inhibitory receptors were decreased, thereby increasing the percentage of cells expressing fewer inhibitory receptors (**Figure 2C**; **Supplementary Figure S2C**). Since inhibitory receptors expression suppresses T cell functions, the reduction in inhibitory receptors expression by byostatin-1 may lead to a restored T cell function by alleviating suppression from inhibitory receptors.

The transcription factors TCF-1 and TOX play central roles in regulating CD8+ T cell exhaustion and their proliferative capacity. TCF-1+ progenitor exhausted T cells maintain effector function, have high proliferative capability and respond to immune checkpoint blockade (ICB) therapy in cancer (43–45), while TOX is sustainably upregulated during T cell exhaustion, and is involved in epigenetic remodeling and transcriptional programming of exhausted CD8+ T cells (46, 47). TCF-1 expression was downregulated in our *in vitro* exhausted CD8+ T cells while TOX was upregulated (**Figures 2D, E**). Bryostatin-1 treatment increased frequency of TCF-1+ repeat peptide stimulated T cells (**Figure 2D**) and decreased TOX expression level in exhausted CD8+ T cells (**Figure 2E**).

Collectively, our data demonstrate that bryostatin-1 reduces exhaustion-related phenotypes in *in vitro*-generated exhausted CD8+ T cells, including a decrease in multiple inhibitory receptors and alterations in key exhaustion-associated transcription factors.

### 2.3 Bryostatin-1 enhances the proliferation and restores the functionality of exhausted CD8+ T cells

**Figure 3:**
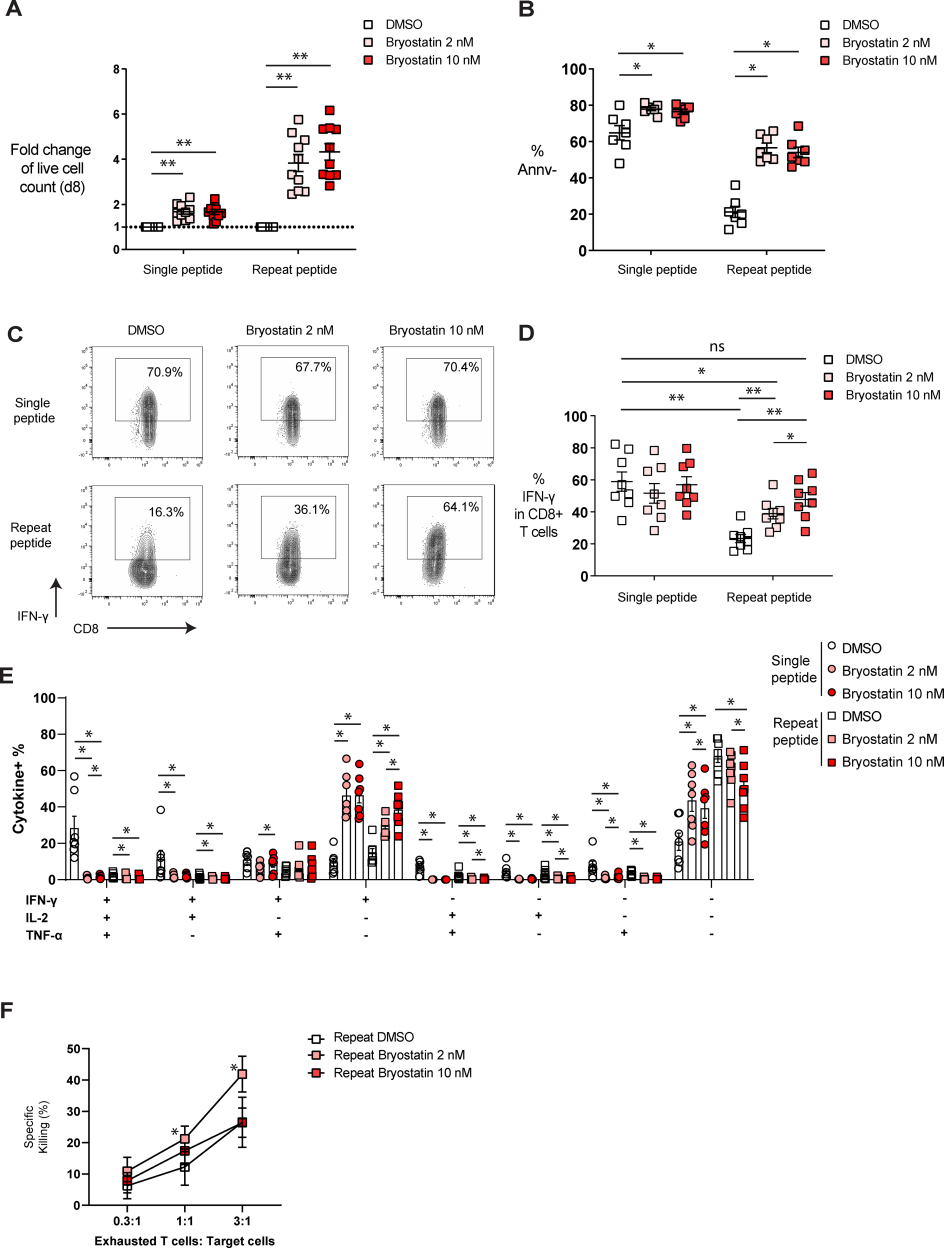
Bryostatin-1 improves the functionality of *in vitro* exhausted T cells. On day 8, cells were either harvested for functional assays or re-stimulated with OVA _(257-264)_ peptide for cytokine production. **(A)** The fold change of live cell counts from both single and repeat peptide-stimulated cells treated with bryostatin-1, compared to the live cell counts from both single and repeat peptide-stimulated cells treated with DMSO on day 8, is shown. **(B)** The frequency of annexin V negative cells (Annv-) depicted for the different culture conditions. **(C)** Representative FACS plots illustrating the frequency of IFN-γ in both single and repeat peptide stimulated cells treated with DMSO or bryostatin-1. **(D)** Pooled data showing the percentage of IFN-γ producing CD8+ T cells from the different culture conditions. **(E)** Bar graph depicting the frequency of cells producing either one, two or three cytokines (IFN-γ, TNF-α, and IL-2). **(F)** Exhausted CD8+ T cells treated either with DMSO, 2 nM or 10 nM bryostatin-1 were co-cultured with target tumor cells at indicated ratios. The percentage of specific killing is depicted. For figure **(A–E)**, each symbol represents one animal (n=7-10), 7 independent experiments were performed. Lines depict mean ± SE. Wilcoxon matched-pairs signed rank test was performed between groups for statistical tests. In figure **(F)**, each symbol represents the mean value from 4 independent experiments and lines depict mean ± SE. Paired t test was performed in figure F to compare the DMSO control and bryostatin-1 treated conditions. ns for not significant, * for p < 0.05 and ** for p < 0.01.

During the progression of T cell exhaustion, antigen-specific CD8+ T cells lose their proliferative capacity, polyfunctionality and cytotoxicity (4, 48). Therefore, we investigated whether bryostatin-1 restores the defective functions of exhausted CD8+ T cells. To assess the proliferative capacity of exhausted cells after bryostatin-1 treatment, we calculated the fold change of cell viability for cells treated either with bryostatin-1 or DMSO from day 5 to day 8. Bryostatin-1 treatment increased the number of live control (single peptide) and exhausted CD8+ T cells on day 8 (**Figure 3A**). This increased number of live CD8+ T cells was accompanied by a trend in enhanced proliferation as both CFSE dilution and the frequency of Ki-67-expressing cells with bryostatin-1 treatment were increased, but this was not statistically significant (**Supplementary Figures S3A, B**). In addition, bryostatin-1 also improved the survival of exhausted CD8+ T cells as the percentage of annexin V negative cells increased upon bryostatin-1 treatment, suggesting that bryostatin-1 may also prevent activation-induced cell death during chronic antigen stimulation (**Figure 3B**).

To examine whether bryostatin-1 modulates the function of exhausted T cells, cells were stimulated on day 8 with OVA_(257-264)_ peptide for 6 hours before cytokine (IL-2, IFN-γ and TNF-α) production was determined by flow cytometry (**Supplementary Figure S4A**). We found that exhausted T cells had fewer IFN-γ-producing cells compared to single peptide-stimulated cells, while bryostatin-1 increased the frequency of IFN-γ-producing exhausted T cells in a dose-dependent manner (**Figures 3C, D**). We then investigated the polyfunctionality of these cells by analyzing the co-production of cytokines in cells. IFN-γ-single producing cells increased in both single and repeat peptide-stimulated cells, showing a dose-dependent effect of bryostatin-1 in mediating the increase of IFN-γ. Frequencies of single IL-2 and TNF-α producing cells were very low in DMSO controls and they decreased further or did not change in non-exhausted control and exhausted CD8+ T cells upon bryostatin-1 treatment (**Figure 3E**; **supplementary Figure S4B**). Cells producing more than one cytokine were also reduced in both, single and repeat peptide stimulated cells (**Figure 3E**). The frequency of dysfunctional exhausted CD8+ T cells that did not produce any of the three cytokines (IFN-γ, TNF-α, and IL-2), decreased after bryostatin-1 treatment (**Figure 3E**). However, single peptide stimulated CD8+ T cells that did not produce any of the three cytokines increased when stimulated with OVA peptide (**Figure 3E**). Furthermore, changes in the cytotoxic capacity of exhausted CD8+ T cells after bryostatin-1 treatment was assessed by killing assay, in which we co-cultured bryostatin-1 or DMSO- treated exhausted OT-I cells with OVA_(257-264)_ peptide pulsed AE-17 mesothelioma cells. Bryostatin-1 at 2 nM increased the percentage of specific killing mediated by exhausted CD8+ T cells (**Figure 3F**). Granzyme B (Gzmb) was significantly downregulated in exhausted T cells after bryostatin-1 treatment, while the expression of CD107a, a marker for degranulation, did not change significantly (**Supplementary Figure S4C**). This may indicate that the improved killing of exhausted T cells by bryostatin-1 is independent of granzyme B-mediated degranulation. Taken together, our data suggest that bryostatin-1 affects exhausted T cells, leading to the restoration of their proliferation, IFN-γ production, and killing capacity.

### 2.4 Bryostatin-1 changes the transcriptional profile of exhausted T cells and upregulates MAPK11

**Figure 4:**
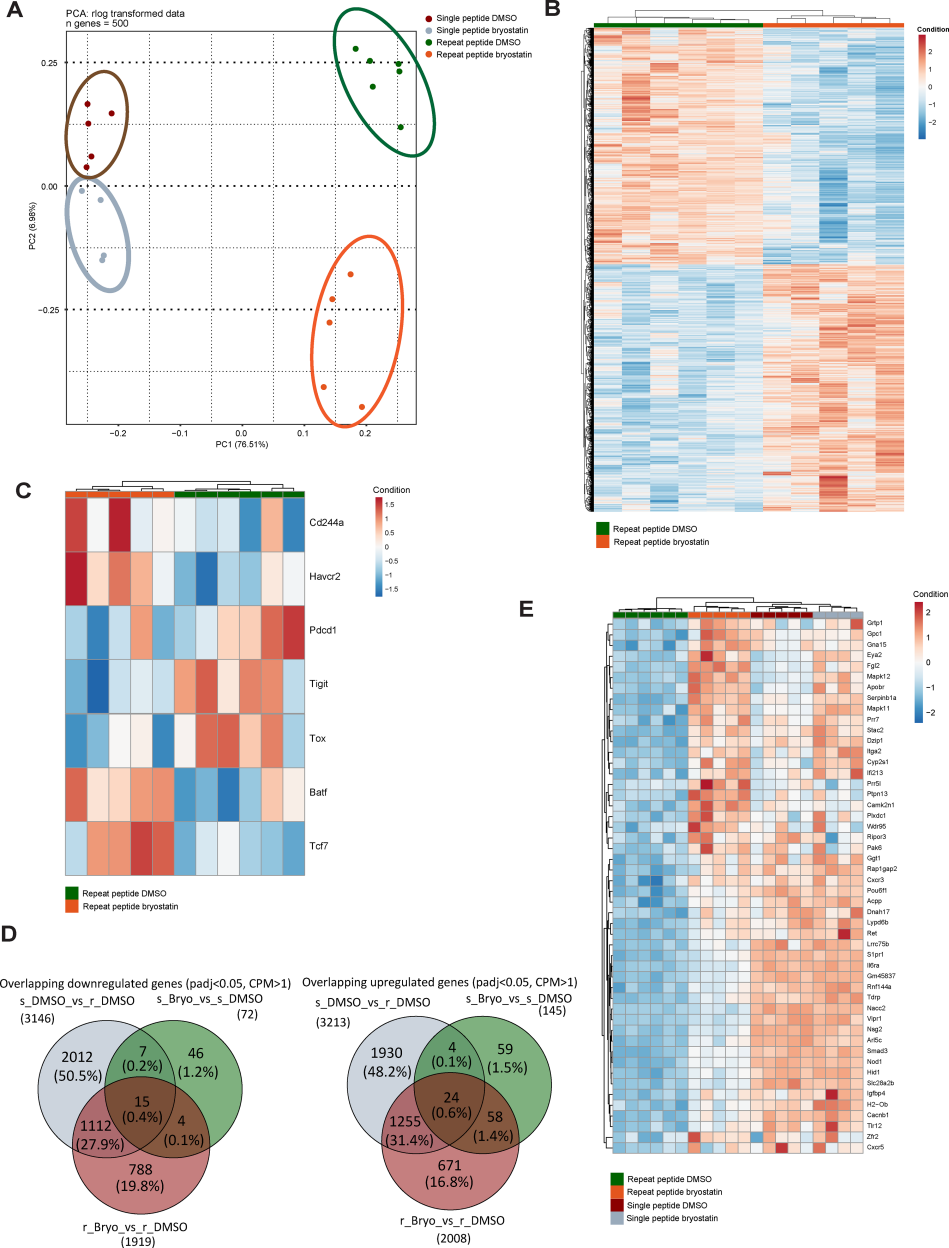
Bryostatin-1 transcriptionally upregulates MAPK 11 in *in vitro* exhausted T cells. RNAseq analysis was performed on both single and repeat peptide stimulated cells which were treated with DMSO or bryostatin-1 for 3 days from day 5 to day 8. **(A)** Principal component analysis (PCA) plot showing the 500 most variable genes on rlog transformed counts. **(B)** Heatmap showing the Z-scores of significantly (adjusted p-value < 0.05, counts per million (CPM) >1) differentially expressed protein-coding genes between bryostatin-1-treated and DMSO-treated exhausted T cells. **(C)** Heatmap showing the Z-scores of a list of selected exhaustion-associate genes which were significantly changed in bryostatin-1 treated exhausted T cells compared to DMSO-treated exhausted T cells. **(D)** Venn diagrams showing the overlap of downregulated DEGs between the three gene sets (left) and the overlap of upregulated DEGs between the three gene sets (right). The three gene sets include comparisons of single peptide DMSO (s_DMSO) vs. repeat peptide DMSO (r_DMSO), single peptide bryostatin-1 (s_Bryo) vs single peptide DMSO (s_DMSO) and repeat peptide bryostatin-1 (r_Bryo) vs repeat peptide DMSO (r_Bryo). **(E)** Heatmap showing the Z-scores of the top 50 differentially expressed genes from the overlapping upregulated genes in the comparisons of single peptide DMSO vs. repeat peptide DMSO and repeat peptide bryostatin-1 vs. repeat peptide DMSO.

To investigate the underlying mechanism of how bryostatin-1 exerts its effects on exhausted CD8+ T cells, we performed RNA-sequencing (RNA-seq) on *in vitro* exhausted OT- I cells which were treated with 10 nM bryostatin-1 or DMSO from day 5 to day 8. As we previously found using this T cell exhaustion culture system (39), principal component analysis (PCA) demonstrated that exhausted CD8+ T cells induced by repeat peptide stimulation were clearly separated from CD8+ T cells from single peptide stimulation (**Figure 4A**). The cluster of bryostatin-1-treated exhausted CD8+ T cells distinctly separated from its counterpart which was treated with DMSO, indicating that bryostatin-1 induced significant gene expression changes in exhausted T cells. Heat maps of differentially expressed genes (DEG), when comparing bryostatin-1 and DMSO-treated exhausted CD8+ T cells, revealed that there were 3927 statistically significant genes (FDR-adjusted p-value < 0.05, counts per million (CPM) >1). Of these, 2009 genes were upregulated and 1918 genes were downregulated in bryostatin-1 treated cells (**Figure 4B**). Gene set enrichment analysis (GSEA) revealed that repeat peptide stimulated cells exhibited the expected exhaustion-associated signatures compared to single peptide stimulated cells (**Supplementary Figure S5A**). However, the gene sets reported to be altered in exhausted T cells were not significantly affected by bryostatin-1 (**Supplementary Figure S5B**). This suggests that bryostatin-1 does not alter the transcriptional program of T cell exhaustion. Further analysis using gene sets representing progenitor or terminally exhausted T cells revealed that neither gene set was affected by brysotatin-1 treatment. This indicates that bryostatin-1 does not significantly alter the proportion of exhausted T cell subpopulations in our system (**Supplementary Figure S5C**). When analyzing RNA-seq data for exhaustion-specific genes, we found that bryostatin-1 treatment significantly reduced the expression of genes encoding inhibitory receptors, such as *Pdcd1* (PD-1) and *Tigit* (**Figure 4C**), showing a fold change of 0.79 and 0.78, respectively. Additionally, bryostatin-1 notably decreased *Tox* expression and substantially increased *Tcf7* expression among exhaustion-related transcription factor genes (**Figure 4C**). The reduction in *Tox* was 0.79-fold, while *Tcf7* increased by 2.5-fold. These changes are consistent with the results obtained by flow cytometry analyses (**Figure 2**). Interestingly, the expression of *havcr2*, which encodes TIM3, was significantly elevated (2.03-fold) following bryostatin-1 treatment although such upregulation was not found in flow cytometry (**Figure 2B**).

We sought to explore the mechanism of byrostatin-1’s effects on exhausted CD8+ T cells. Therefore, we performed further analysis on genes associated with exhaustion that are affected by bryostatin-1 in RNA-seq data. We focused on the top 50 significantly DEGs ranking by log_2_FoldChange within two principal groups: genes which are upregulated in exhausted CD8+ T cells and downregulated by bryostatin-1 (1127 genes), and genes downregulated in exhausted CD8+ T cells but restored by bryostatin-1 (1279 genes) (**Figure 4D**). Among the top 50 of 1279 genes, we found that the p38 MAPK family member MAPK11 (p38 β), which was downregulated in exhausted CD8+ T cells, was upregulated after bryostatin-1 treatment by 3.15 fold (**Figure 4E**). Since p38 mitogen activated protein kinases (MAPK) are located downstream of PKC and the activation of p38MAPK signaling has been associated with cell proliferation and cytokine expression of T cells (49, 50), we hypothesized that MAPK11 modulated by bryostatin-1 may be regulating the downstream signaling of exhausted T cells. Another p38 MAPK family member, MAPK12 (p38 γ), also showed a similar trend as MAPK11, however, it has low basal expression in T cells. MAPK13 (p38 δ) is not expressed in T cells, with no detectable transcripts observed in RNA-seq. MAPK14 (p38 α) is not directly associated with exhaustion signature of T cells, as its expression does not show significant changes in repeat peptide-stimulated T cells treated with DMSO compared to single peptide-stimulated T cells treated with DMSO in RNA-seq, though upregulated by bryostatin-1. Thus, we decided to focus on how upregulated MAPK11 contributes to the effect of bryostain-1 on exhausted CD8+ T cells. It has been reported that blocking p38 MAPK inhibited bryostatin-1 and IL-2 induced IFN-γ production in human primary T cells (51), suggesting that bryostatin-1 upregulated MAPK11 in exhausted CD8+ T cells may play an important role in improving phenotypic and transcriptional profile of exhausted CD8+ T cells.

### 2.5 MAPK11 inhibitor partially blocks bryostatin-1 effects in *in vitro* exhausted CD8+ T cells

**Figure 5:**
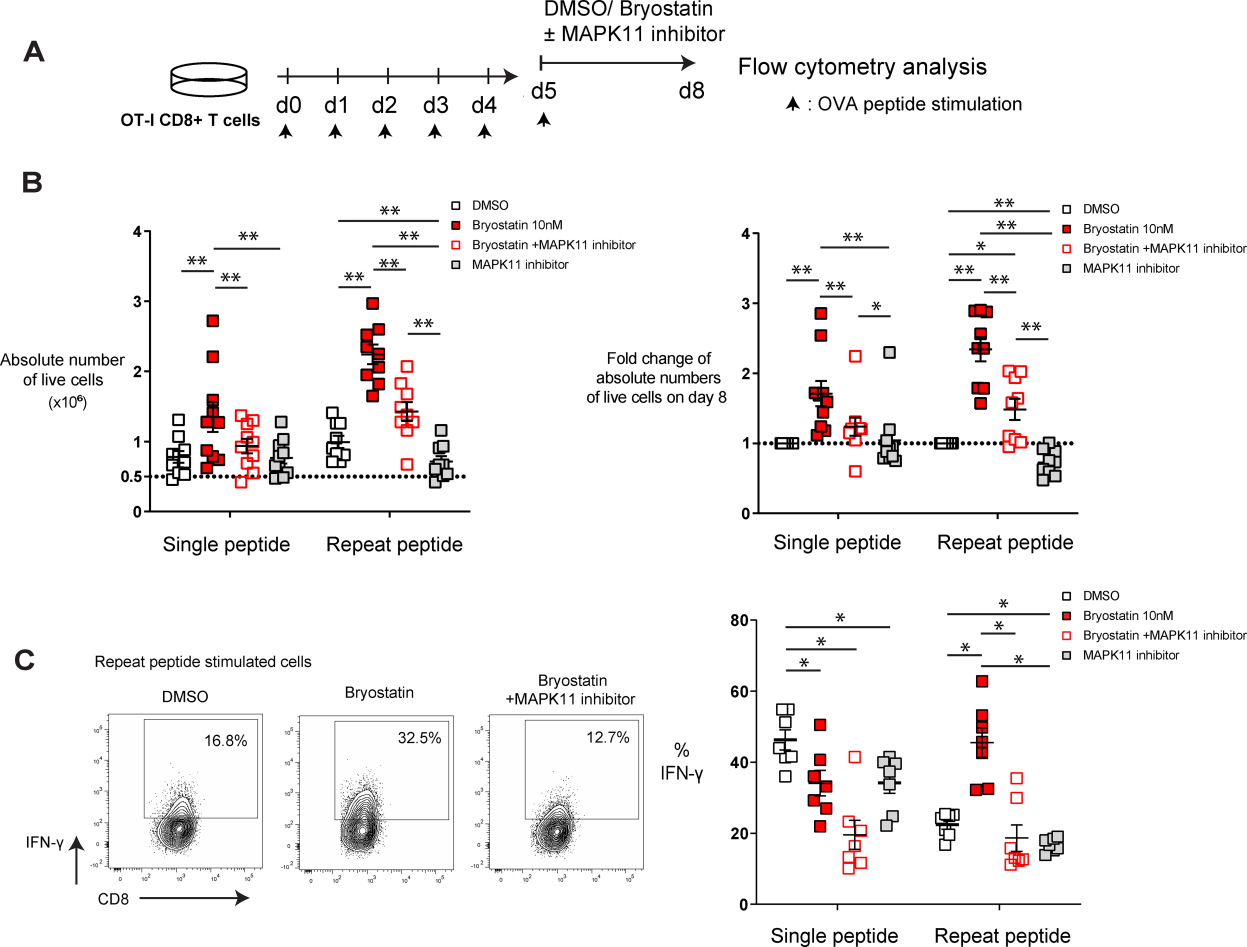
MAPK 11 inhibition blocks bryostatin-1-medidated cell proliferation and IFN-γ production in *in vitro* exhausted T cells. *In vitro* generated exhausted T cells (repeat peptide stimulated cells) and non-exhausted T cell controls (single peptide stimulated cells) were treated with bryostatin-1 or DMSO concurrent with or without RWJ-67657 for 3 days from day 5 to day 8. **(A)** Scheme of testing bryostatin-1 or DMSO concurrent with or without MAPK 11 inhibition in an *in vitro* exhaustion assay. **(B)** Absolute numbers of live cells (left) for indicated treatment condition is depicted. Fold change of absolute numbers of live cells in bryostatin-1 or/and MAPK11 inhibitor treated condition compared to DMSO control on day 8 is shown. **(C)** Representative FACS plots illustrating the frequency of IFN-γ producing CD8+ T cells within exhausted T cells treated with bryostatin-1 in the presence/absence of RWJ-67657 (left). Pooled data showing the percentage of IFN-γ producing CD8+ T cells from the different culture conditions (right). Each symbol represents one animal (n=7-9), 5 independent experiments were performed. Lines depict mean ± SE. Between groups, Wilcoxon matched-pairs signed rank test was performed for statistical tests. * for p < 0.05, ** for p < 0.01.

To investigate whether the upregulated MAPK11 in exhausted T cells contributes to the beneficial effects of bryostatin-1, we utilized RWJ-67657, a p38α/β inhibitor, in our *in vitro* exhaustion system to block the activation of MAPK11. As above, CD8+ T cells were exhausted on day 5 after repeat peptide stimulation and from day 5 to day 8 cells were either treated with 10 nM bryostatin-1 or DMSO combined with RWJ-67657 (**Figure 5A**). RWJ-67657 decreased the cell numbers induced by bryostatin-1 but did not affect cell death of bryostatin-1 treated exhausted CD8+ T cells (**Figure 5B**; **Supplementary Figure S6A**), suggesting that RWJ-67657 blocked the bryostatin-1-induced increase in proliferation of exhausted CD8+ T cells. On day 8, cells were stimulated with OVA_(257-264)_ peptide for 6 hours and assessed for cytokine production. We found that RWJ-67657 abrogated bryostatin-1’s enhanced IFN-γ production of exhausted CD8+ T cells (**Figure 5C**). However, the decrease in inhibitory receptors induced by bryostatin-1 was not blocked by RWJ-67657 (**Supplementary Figure S6B**), indicating that this decrease is not dependent on the activation of MAPK11.

Taken together, these data indicate that bryostatin-1 specifically improves proliferation and IFN-γ production of exhausted T cells in a MAPK11-dependent manner. However, downregulation of inhibitory receptors by brytostatin-1 on exhausted CD8+ T cells is independent MAPK11 signaling.

## 3 Discussion

Bryostatin-1 has been studied for HIV therapy and cancer treatment due to its broad pharmacological effects via PKC isozyme modulation (8, 29, 52). It reactivates latent HIV in cellular models of HIV latency and exhibits significant immunomodulatory antitumor activity both *in vitro* and *in vivo* (16, 19–21). CD8+ T cells, crucial defenders against viral infected and malignant cells, become exhausted with chronic antigen exposure, leading to functional decline (2, 3, 48). In this study, we found that bryostatin-1 improved dysfunctional HIV-specific CD8+ T cell proliferation. Using an *in vitro* exhaustion assay, which allows us to study how pharmacological compounds directly act on exhausted T cells, we show that bryostatin-1 directly reduced exhausted-associate markers and improved exhausted CD8+ T cell functions, including proliferative capacity, IFN-γ production and cytotoxicity. The improved proliferation and IFN-γ production of exhausted CD8+ T cells was dependent on the activation of MAPK11 by bryostatin-1.

Bryostatin-1 is an HIV inhibitor and a latency-reversing agent that has been studied in preclinical and clinical studies (30–32). It is expected that it could contribute to the “shock and kill” strategy by reactivating latent HIV, making infected cells visible to CD8+ T cells, which then recognize and eliminate them (33). Therefore, investigating how bryostatin-1 influences CD8+ T cells in HIV patients is essential for optimizing the immune response and enhancing the overall effectiveness of the “kill” phase in this approach. In our study, we enrolled fifteen PWH who received antiretroviral therapy (ART) and had undetectable plasma viral loads. Bryostatin-1 itself is not mitogenic to T cells (20, 53, 54) and requires additional factors that together with the activation of PKC to achieve T cell activation, such as a calcium ionophore (20), cytokines like IL-2 (55), or costimulatory signals such as CD28 (40). These activation signals are independent of TCR stimulation. We found that bryostatin-1, along with the CD28 costimulatory signal, vigorously expands T cells in PBMCs from PWH, including both CD4+ and CD8+ T cells. Notably, the proportion of HIV-specific CD8+ T cells preferentially increased after byostatin-1 treatment, indicating that they may be expanded more during culture compared to non-specific T cells. As the cellular signaling transduction, including basal phosphorylation levels of proteins in the pathways downstream of the T cell receptor (TCR), changes in T cells from PWH at different stages of disease progression (56), HIV-specific CD8+ T cells may exhibit pre-existing TCR signaling activity compared to quiescent naïve CD8+ T cells. This may lead to the increased expansion of HIV-specific CD8+ T cells and is consistent with previous findings by Chin et al. that bryostatin-1 preferentially expands tumor-specific cells that have been exposed to tumor-cell antigens, rather than naïve cells (23). Bryostatin-1 is an HIV latency-reversing agent, which can reactivate the virus from HIV reservoirs. Given the fact that the proportion of HIV-latently infected cells in patients is exceptionally low and the fraction of cells with replication-competent HIV-1 proviruses is even smaller, approximately 0.1–10 cells per million in resting CD4+ T cells (57–60), the likelihood of releasing HIV antigens from HIV-latently infected cells is extremely low. This is because in our culture system we cultured 10^6^ PBMCs per well, with only 40–50% of these being CD4+ T cells and CD8+ T cells present, making the production of virus unlikely. Therefore, the increased proliferation of HIV-specific CD8+ T cells by bryostatin-1 is most likely independent of antigen stimulation, although this cannot be completely excluded.

Bryostatin-1 promoted the proliferation of both CD4+ and CD8+ T cells in our cultures. Previously, we compared the cytotoxic effects of bryostatin-1 on CD4+ and CD8+ T cells after a 3-day treatment (35). We found that bryostatin-1 did not induce significant drug-specific cell death in unactivated or activated CD8+ T cells (35). However, unactivated CD4+ T cells exhibited reduced survival at bryostatin-1 concentrations of 10 nM and above (35), suggesting that CD4+ T cells may have differing sensitivity to bryostatin-1 when compared to CD8+ T cells. In another study, it was reported that bryostatin-1, in combination with IL-2, increases IFN-γ mRNA expression in human peripheral blood CD4+ and CD8+ T cells (51), suggesting that subsets of T cells may respond similarly to bryostatin-1 in terms of functional changes. In the current study, we focused primarily on exhausted CD8+ T cells, with a particular emphasis on HIV-specific CD8+ T cells. Since HIV-specific CD8+ T cells control virus (61), despite reduced functionality and impaired proliferation during chronic infection (36, 62), expanding them is expected to improve immune control over HIV once the virus is reactivated. Our data expand the understanding of bryostatin-1’s effect on HIV-specific T cell immunity, as it is being studied as a candidate latency-reversing agent. Although a completed phase I clinical trial with bryostatin-1 did not induce detectable activation of HIV RNA transcription in ART-treated HIV-infected individuals (32), this may be explained by the fact that the highest plasma concentration achieved in these patients after a single dose of 10 or 20 μg/m² was well below nanomolar levels which were required for *ex vivo* HIV reactivation (32, 63, 64). This suggests that further investigation is needed to evaluate bryostatin-1 in the clinical setting. Since bryostatin-1 broadly activates T cells in the presence of costimulatory signals or cytokines like IL-2, it may suggest a risk of triggering auto-reactive response while exerting its beneficial anti-tumor effects. Additionally, bryostatin-1 induces the production of proinflammatory cytokines and induces *in vivo* plasma levels of IL-6 and TNF-α (65, 66). However, no clinical trials to date have reported auto-reactive side effects with bryostatin-1 (24–28).

PD-1 expression is upregulated on HIV-specific CD8+ T cells in both viremic and aviremic patients, and associates with impaired effector function of these cells (36, 67). We found that bryostatin-1 significantly decreased PD-1 levels on HIV-specific CD8+ T cells, while retaining their activation as indicated by increased CD38 expression. In our study, the HIV-infected patients were all virologically suppressed by ART treatment. Compared to the cells in untreated rapid progressors or chronic HIV+ progressors, HIV-specific CD8+ T cells after ART tend to be less exhausted as they have lower PD-1 expression and improved proliferative capacity (36, 68, 69). However, the polyfunctionality, proliferative and killing capacities of HIV-specific CD8+ T cells in patients treated with ART is not restored to the levels observed in long-term nonprogressors (LTNP), despite low antigen levels following ART (70).

Our *in vitro* exhaustion system enables us to examine the effect of bryostatin-1 directly on CD8+ T cell exhaustion, a state that occurs in the context of chronic viral infection and tumors. Bryostatin-1 treated exhausted CD8+ T cells showed enhanced proliferation and decreased apoptosis. Similarity, we also observed that bryostatin-1 improved the expansion and survival in single peptide stimulated CD8+ T cells in the absence of antigen, indicating that bryostatin-1 induces proliferation of T cells independent of antigen stimulation. Previous reports indicated that additional supplementation of exogenous IL-2 is necessary to ensure T cell survival and expansion during activation by bryostatin-1 (55). In our culture conditions we include IL-7 and IL-15, both belonging to the common γ chain cytokine family, and known to have anti-apoptotic potential compared to IL-2 (71, 72). Our data are consistent with previous findings showing that IL-7 and IL-15 increase the yield of T cells activated with bryostatin-1 and ionomycin (B/I), suggesting that IL-7 and IL-15 may synergize with bryostatin-1 in our observed effects (73). Using our *in vitro* exhaustion system, we found that bryostatin-1 improved CD8+ T cell exhaustion phenotypically and functionally, including reducing exhaustion-associated markers on cell surface while increasing killing and IFN-γ production. In keeping with our previous studies, we find that our *in vitro* cultured exhausted T cells recapitulate the transcriptomic characteristics of CD8+ T cells from LCMV clone 13 infection (39). However, we did not observe that bryostatin-1 significantly influenced the transcriptional program associated with T cell exhaustion. Specifically, transcriptional changes by bryostatin-1 did not affect gene sets linked to T cell exhaustion, including the predefined exhaustion gene signatures of progenitor exhausted and terminally exhausted T cell subsets. One limitation of our current study is the absence of *in vivo* validation of these finding due to the limited number of cells obtainable from *in vivo* exhaustion models or HIV-infected patient samples. Further investigations using *in vivo* exhausted T cells from treated animals, particularly focusing on differential effects directly on progenitor and terminally exhausted subsets, will provide deeper mechanistic insights of bryostatin-1 activity.

Bryostatin-1 has been used to stimulate T cells in cancer immunotherapy as T cells from tumor-sensitized lymph nodes treated with bryostatin-1 and ionomycin (B/I) exhibit enhanced control of tumor progression after adoptive transfer into tumor-bearing mice (22, 23, 74, 75). This could be explained by the finding that B/I preferentially activate CD62L^low^ CD8+ T cells, which are enriched in tumor antigen-specificity, compared to their naive CD62L^High^ counterparts in lymph nodes harvested from mice with tumors (23). In our studies, we found that exhausted CD8+ T cells treated with a low concentration (2 nM) of bryostatin-1 showed an increased cytotoxicity, though there was no improvement on exhausted CD8+ T cell cytotoxicity when cells were treated with high concentration of bryostatin-1 (10 nM). This suggests that bryostatin-1 may control T cell cytotoxicity at different concentrations by distinct mechanisms other than granzyme B-mediated degranulation that affect activation, cell-to-cell contact or non-degranulation mechanisms, such as FasL and TRAIL. As reported, bryostatin-1 impacts cytokines production. Bryostatin-1 induced proinflammatory cytokines such as IL-8, IL-1β, TNF-α, and IL-6 in human monocytes and increased TNF-α secretion of chronic myelomonocytic leukemia cells (65, 76). Six hours treatment of bryostatin-1 increased the production of both TNF-α and IFN-γ, and IL-2 following anti-CD3/CD28 stimulation in elite suppressor HIV-specific CD8 + T cells (77). Bryostatin-1 induces both IFN-γ mRNA and protein expression in human T cells in the presence of IL-2 (51). Our findings showed that bryostatin-1 only enhanced IFN-γ single producing cells in exhausted CD8+ T cells when restimulated with peptide, while having no impact on, or decreasing, TNF-α and IL-2 production. Furthermore, in single peptide stimulated T cells it inhibited polyfunctionality and increased IFN-γ single producing cells. This is consistent with the previous finding that bryostatin-1 blocks the IL-2 secretion in activated T cells from tumor-draining lymph nodes (55). The decreased IL-2 production suggests that exogenous IL-2 may need to be administered simultaneously with bryostatin-1 to achieve optimal anti-tumor activity (55). Additionally, the reduction in TNF-α production in T cells by bryostatin-1 may be compensated by its ability to increase TNF-α production from macrophages (78), something that would promote anti-tumor activity within the tumor microenvironment. Given that PKC mediates posttranscriptional regulation of cytokine production in CD8+ T cells and bryostatin-1 can either activate or inhibit PKC depending on the duration of incubation (8, 79), the varied effects of bryostatin-1 on cells may be attributed to differences in treatment duration and cell type.

Through the RNA-seq analysis, we found that MAPK11 (p38β) was significantly downregulated in exhausted CD8+ T cells and was significantly upregulated upon bryostatin-1 treatment. MAPK11 belongs to the p38 MAPK family. P38 together with extracellular response kinases (ERK) and c-Jun amino terminal kinases (JNK) are members of the MAP kinase family. PKC can activate p38 MAPK (80) which in turn mediates T cell proliferation, apoptosis and cytokine production (49, 81, 82). By using the pharmacological agents RWJ-67657, which blocks MAPK11, we found that the increased proliferative capacity of both exhausted and non-exhausted CD8+ T cells mediated by bryostatin-1 was blocked. Furthermore, the bryostatin-1-mediated increased IFN-γ production by exhausted CD8+ T cells was also blocked by MAPK11 inhibition. This is consistent with previous reports that activation of p38 MAP kinase in CD8+ T cells increases IFN-γ production (49, 83) and that inhibition of p38 MAPK blocks T cell proliferation (82) and IFN-γ production (51). However, RWJ-67657 did not change bryostatin-1 induced downregulation of inhibitory receptors, which may be regulated in a p38 MAPK-independent manner. Taken together, our data showed that inhibition of MAPK11 partially abrogated the effects of bryostatin-1 on exhausted CD8+ T cells.

In summary, we show here that bryostatin-1 enhances cell proliferation and reduces PD-1 expression of HIV-specific CD8+ T cells. Bryostatin-1 directly reduces exhausted-associate markers and improves proliferative capacity, IFN-γ production and cytotoxicity of *in vitro* generated exhausted CD8+ T cells. MAPK11 inhibition blocks the increased proliferation and IFN-γ production induced by bryostatin-1. These data suggest that bryostatin-1 may potentially enhance CD8+ T cell responses by targeting exhausted CD8+ T cells in the treatment of infections insufficiently controlled by available therapies, to help in eradicating HIV reservoir cells, or by improving the effectiveness of cancer immunotherapy.

## 4 Material and methods

### 4.1 PBMCs from PWH and ethical approval

Fifteen people living with HIV (PWH) were included in this study after approval from the Erasmus MC Medical Ethics Committee (MEC-2012–583, METC-2016-076) and receiving written informed consent. Fourteen patients received ART during chronic HIV infection and achieved effective viral suppression below 50 copies/ml for at least a year, with one patient having an undetectable viral load for at least 6 months, though it may have been suppressed longer. Peripheral blood mononuclear cells (PBMC) were isolated from whole blood by Ficoll-Paque density gradient centrifugation and cryopreserved for subsequent testing.

C57BL/6 Tg (TcraTcrb)1100Mjb/J (OT-I) mice were originally purchased from Charles River France and were bred in-house. OT-I mice were genotyped by PCR and flow cytometry analysis before they were used in experiments. Mice were housed in ventilated cages with a maximum density of four mice per cage at the Erasmus Medical Center animal facility (Erasmus Dierenexperimenteel Centrum, EDC), with food and water provided ad libitum. All animal studies were performed under ethical approval by the Instantie voor Dierenwelzijn (IvD) and project license (IRN# 2020-0014 CCD# 209604) approved by Centrale Commissie Dierproeven (CCD).

### 4.2 Cell proliferation assays

For the human PBMC proliferation assay, PBMCs from PWH were labeled with the CellTrace™ Far Red Cell Proliferation Kit (Thermo Fisher, C34564). Human PBMCs were spin down after thawing, and incubated in 100 nM cell trace dye solution for 20 minutes at 37°C in the dark. The reaction was stopped by adding 5× volume RPMI medium (containing 10% FBS) and incubated for 5 minutes to quench the unreacted dye at 37°C. Cells were cultured in RPMI 1640 medium (containing 10% FBS, 2mM L-glutamine, 100 U/ml penicillin and 100 μg/ml streptomycin-sulfate) with bryostatin-1 (sc-201407, Santa Cruz Biotechnology) or DMSO for 6 days. The concentration of bryostatin-1 was determined based on previous findings, where 10 nM bryostatin-1 significantly downregulated PD-1 expression on CD8+ T cells from PWH (35). Lower concentrations of bryostatin-1, ranging from 0.1 nM to 10 nM, were tested on four out of the fifteen PBMC samples. All samples were cultured with 1 μg/mL of anti-CD28 and anti-CD49d antibodies (NA/LE anti-CD28 clone CD28.2, anti-CD49d clone 9F10; BD Biosciences). On day 6, cells were harvested for flow cytometry staining and analysis.

For the proliferation assay of murine CD8+ T cells, single peptide and repeat peptide stimulated cells were cultured from day 0 to day 5 as described below. On day 5, both single peptide and repeat peptide stimulated cells were washed once and re-suspended in 0.1 µM CellTrace™ CFSE Cell Proliferation dye solution (Thermo Fisher, C34554). Cells were incubated at 37°C for 20 minutes in the dark. To stop the reaction, a 5× volume of RPMI medium (containing 10% FBS) was added, and the cells were incubated for an additional 5 minutes at 37°C to quench the unreacted dye. After CFSE labeling, single peptide and repeat peptide stimulated cells were cultured for an additional 3 days in the presence of either bryostain-1 or DMSO. On day 5, 10 ng/ml OVA_(257-264)_ peptide was added to repeat peptide stimulation cultures. On day 8, cells were harvested for flow cytometry staining and analysis.

### 4.3 *In vitro* murine T cell exhaustion induction and drug treatment

Exhausted CD8+ T cells were induced by repeat OVA_(257-264)_ peptide stimulation as previously reported (39). As described, CD8+ T cells were isolated from single-cell suspensions of splenocytes from OT-I mice by negative selection (EasySep, Stemcell Technologies). Cells were cultured in RPMI 1640 media containing 10% FBS (Gibco), 2 mM L-glutamine (Life Technologies), 10 mM HEPES (Life Technologies), 100 mM Sodium Pyruvate (Life Technologies), 1% non-essential amino acides (Gibco), 100 U/ml penicillin (Gibco) and 100 μg/ml Streptomycin-sulfate (Gibco), 0.05 mM Betamercaptoethanol (Sigma). Cytokines IL-7 (5 ng/ml, Peprotech, Cat 210–07) and IL-15 (5 ng/ml, Peprotech, Cat 210–15) were added in the media to avoid cell apoptosis. Cells received repeat OVA_(257-264)_ peptide stimulation daily at the concentration of 10 ng/ml for five days from day 0 to day 5 which inducing T cell exhaustion. The cells received only a single peptide stimulation on day 0 were washed after 48 hours, resulting in a non-exhausted effector phenotype. On day 5, both single peptide and repeat peptide stimulated cells were treated either with 2 nM bryostatin-1, 10 nM bryostatin-1 or DMSO for additional 3 days. 10 ng/ml OVA_(257-264)_ peptide was added to repeat peptide stimulation cultures to maintain the chronic antigenic stimulation environment. For blocking MAPK11 activation, 10 μM RWJ-67657 was used concurrently with 10 nM bryostatin-1 from day 5 to day 8. The concentration of RWJ-67657 (Biotechne) was determined based on drug titration (from 0.1 μM to 100 μM).

On day 8, cells were stained by 0.4% trypan blue solution (Gibco) and counted using an automated counter (Countess, Life Technlogies). Cells were then stained for flow cytometry.

### 4.4 Flow cytometry

For staining PBMCs from PWH, cells were washed with FACS wash (HBSS containing 3% FBS and 0.02% sodium azide) and incubated with fluorochrome-conjugated monoclonal antibodies at 4°C in the dark for 30 minutes. Antibodies used were anti-CD3-BV510 (SK7, Biolegend), anti-CD3- BV421 (UCHT1, BD Biosciences), anti-CD4-APC-Cy7 (SK3, Biolegend), anti-CD4-BV650 (SK3, BD Biosciences), anti-CD8- BV786 (RPA-T8, BD Biosciences), anti-PD-1-BV711 (EH12.1, BD Biosciences), and anti-CD38-PE-cy7 (HIT2, BD Biosciences). To analyze HIV-specific CD8+ T cells, in house prepared tetramers of HLA class I A*0201 labeled with PE or PE-Cy7 and loaded with either HIV-Gag p17 77-85 (SLYNTVATL or HIV-Pol 476-484 (ILKEPVHGV were used. PerCP/Cyanine5.5 labeled Annexin V (BD Biosciences) was used to exclude apoptotic and dead cells. Annexin V stains were performed in the presence of 2.5mM CaCl_2._ After staining, cells were washed twice with FACS wash and then fixed with 1% Paraformaldehyde solution (PFA) containing 2.5 mM CaCl_2_.

For surface staining of single or repeat peptide stimulated murine CD8+ T cells, cells were firstly washed with FACS wash and then incubated at 4°C in the dark for 20 minutes with previously determined concentrations of fluorochrome-conjugated monoclonal antibodies: anti-CD8a-eFluor 450 (clone 53-6.7, eBioscience), anti-CD160-PE-CF594 (clone CNX46-3, BD Biosciences), anti-LAG-3-APC (clone C9B7W, BD Biosciences), anti-CD244-PE (clone 2B4, BD Biosciences; eBio244F4, eBioscience), anti-PD-1-APC-Cy7 (clone 19F.1A12, Biolegend), anti-TIM-3-PE-Cy7 (clone RMT3-23, Invitrogen), and anti-TIGIT-FITC (clone GIGD7, eBioscience). PerCP/Cyanine5.5 conjugated Annexin V (BD Biosciences) was used to exclude apoptotic and dead cells. After incubating, cells were washed one time with FACS wash and fixed with 1% PFA. All staining steps contained 2.5 mM CaCl_2_.

For the intranuclear staining of Ki-67 and transcription factors, cells were first surface stained as described above. After washing with FACS wash, cells were fixed with FoxP3 Fixation Buffer (catalogue # 005523, eBioscience) for 1 hour in the dark at 4°C. Cells were then washed with Perm/Wash buffer (catalogue # 008333, eBioscience) and stained at 4°C in the dark with antibodies against transcription factors anti-Tox-PE (clone TXRX10, eBioscience), and anti-TCF1-A647 (clone C63D9, Cell Signaling) and anti-Ki-67- Alexa Fluor^®^ 700 (16A8, Biolegend). After 45 mins, cells were washed twice with Perm/Wash buffer and fixed with 1% PFA.

To detect cytokine production, single or repeat peptide stimulated murine CD8+ T cells were re-stimulated with 10 µg/ml OVA_(257-264)_ SIINFEKL peptide for 6 hours at 37°C, 5% CO_2_ in the presence of GolgiPlug (BD Biosciences). Anti-CD107a-APC-Cy7 antibodies (clone ID4B, Biolegend) was also added during 6 hours culture if it was examined. After 6 hours, cells were then stained with surface antibody anti-CD8a-eFluor 450 (clone 53-6.7, eBioscience), PerCP/Cyanine5.5 conjugated Annexin V (BD Biosciences) and anti-CD107a-APC-Cy7 (clone ID4B, Biolegend). After washing with FACS wash, cells were fixed with IC Fixation Buffer (88-8824, eBioscience) at 4°C overnight, and then washed with Perm/Wash buffer. Cells were stained for intracellular cytokines for 45 minutes in the dark at 4°C with antibodies: anti-IFN-γ-APC (clone XMG1.2, eBioscience), anti-TNF-α-AF488 (clone MP6-XT22, eBioscience), anti-IL-2-PE (clone JES6-5H4, eBioscience) and anti-granzyme B-PE-Cy7 (clone NGZB, eBioscience). After staining, cells were washed twice with Perm/Wash buffer and fixed with 1% PFA.

All samples were measured within 48 hours after fixation. All washing and fixation buffers included 2.5 mM CaCl_2_ to ensure Annexin V binding. Samples were measured on a LSRFortessa (BD Biosciences) using application settings. At least 100,000 events per sample were collected. Data were analyzed with FlowJo software (Version 9.9.4, Treestar, Ashland, OR, USA).

### 4.5 T cell killing assay

To examine the killing capacity of exhausted CD8+ T cells, *in vitro* generated exhausted CD8+ T cells were co-cultured with AE-17 mesothelioma cell line. For tumor cells to serve as target cells recognizable by OT-I T cells, AE-17 cells were resuspended with cell culture medium (RPMI 1640 with 10% FBS) at 10^6^ cell/ml, then pulsed with 1 μg/ml OVA_(257-264)_ peptide for 1 hour at 37˚C. These OVA_(257-264)_ peptide pulsed cells were then washed and labelled with CellTrace™ Far Red fluorescent dye (Invitrogen™, C34564). For tumor cells which serve as non-target control cells, un-pulsed AE-17 cells were either labelled with CellTrace™ CFSE fluorescent dye or without labelling (Invitrogen™, C34554). During co-culturing, 10^5^ peptide-pulsed and 10^5^ un-pulsed AE-17 cells were seeded in 12-well plates and co-cultured with different ratios of exhausted T cells (Effector: Target ratio: 0.3, 1:1, 3:1) for 18 hours. After 18 hours, cells were harvested and stained with anti-CD8a-eFluor 450 antibody (clone 53-6.7, eBioscience) and PerCP/Cyanine5.5 conjugated Annexin V (BD Biosciences) for flow cytometry analysis. The percentage of specific killing of T cells was calculated by the formula: % specific killing = 100 –[Target/Non-target (in the presence of effector cells)]/[Target/Non-target (in the absence of effector cells)] x 100%.

### 4.6 RNA sequence analysis

Using the *in vitro* T cell exhaustion system, single peptide stimulated and repeat peptide stimulated cells were treated from day 5 to day 8 with DMSO or 10 nM bryostatin-1 as described above. On day 8, sorted 0.5-1*10^6^ live CD8+ T cells (Annexin V- CD8+) were washed with PBS and immediately lysed with TRIzol LS reagent (Life Technologies). Samples were stored at -80°C before RNA extraction. RNA isolation was carried out according to the manufacturer’s instructions for TRIzol LS reagent. The Agilent Bioanalyzer^®^ RNA 6000 Nano/Pico Chip was used to determine the quality and quantity of the extracted RNA. Barcoded sequencing libraries were prepared using the NEBNext Ultra II Directional RNA Library Prep Kit for Illumina (NEB #E7760S/L). Briefly, mRNA was isolated from total RNA using oligo-dT magnetic beads, followed by mRNA fragmentation and cDNA synthesis. The cDNA was ligated with sequencing adapters and subjected to PCR amplification. Quality and yield of the prepared samples were assessed using the Fragment Analyzer (Agilent), confirming a broad peak between 300-500 bp in size, consistent with the expected distribution. Paired-end sequencing was performed on a Hiseq2500 machine (Illumina) for 150 cycles.

Paired-end raw FASTQ files were analyzed with the nf-core/RNA-seq pipeline (v3.0) using Nextflow (v20.11.0-edge) and its default settings (84, 85). Within this pipeline quality of the sequencing was reported with FastQC (v0.11.9). Subsequently, low quality bases (Phred scores <= 30) were trimmed using Trim Galore! (v0.6.6). Trimmed FASTQ reads were mapped to the mouse reference genome version GRCm38 with Ensembl GRCm38.81.gtf gene annotation file using RSEM (v1.3.1), which umbrellas STAR (v2.7.6a) as read aligner. Next, SAMtools (v 1.10) processed the alignment files and extracted mapping statistics of the post-alignment for quality control purposes. The quality of each sample alignment was visually inspected using reports derived from RSeQC (v3.0.1), Qualimap (v2.2.2-dev) and Preseq (v2.0.3), including read inner distance plots, splice junction annotations, the genomic origin of the mapped reads, and the estimated complexity of the sequencing library. RSEM estimated transcript counts were imported into R (v4.4.1), transformed to gene counts using tximport (v1.32) and analyzed with DESeq2 (v1.44.0) (86, 87). Only “protein_coding” genes were selected for differential expression analysis. Gene counts were transformed for visual inspection using the “rlog” function of DESeq2 and for Principal Component Analysis (PCA) utilizing its “plotPCA” function.

### 4.7 Differential expression analysis

Differentially expressed genes were calculated using DESeq2 (v1.44.0). p-values were calculated using Wald statistical test and independent hypothesis weighting was applied. Fold Changes were shrunk with the DESeq2 function “lfcshrink” using method “ashr”. Only genes with more than one count per million (CPM) in the protein-coding gene list were considered for further analysis. Genes were indicated as differentially expressed with an adjusted p-value < 0.05, calculated with the Benjamini-Hochberg multiple hypothesis testing method. Heatmaps were made using the R package pheatmap (v1.0.12), Z-scores were calculated per gene on the rlog transformed counts. Hierarchical clustering was performed using complete clustering on the Euclidean distances for both the samples and genes using complete linkage method. Venn diagrams were generated using Excel based on the list of differentially expressed genes.

### 4.8 Gene Set Enrichment Analysis

Gene Set Enrichment Analysis (GSEA) was performed with the protein coding genes after shrinkage of the fold changes and after CPM filtering. Genes were ranked on the fold changes, high to low, and compared with CD8+ T cell specific exhaustion gene-sets. The CD8+ T cell exhaustion gene-sets were derived from Bengsch B, et al. (88) and downloaded from PubMed Central. The GSEA was calculated separately for the upregulated and downregulated on these CD8+ T cell exhaustion gene-set genes. Gene sets described in progenitor and terminally exhausted T cells from LCMV Clone 13 were downloaded from Miller BC, et al. (89). Enrichment plots were made using R package fgsea (v1.18.0) with 100.000 permutations.

### 4.9 Statistics

For flow cytometry analysis, statistical analysis was conducted using Prism software (GraphPad Prism 9, Version 9.0.0). Wilcoxon matched-pairs signed rank test was employed to compare the effect of bryostatin-1 on HIV-specific CD8+ T cells and *in vitro* generated murine exhausted CD8+ T cells. Paired t test was performed after checking normality with the Shapiro-Wilk test, as indicated in the figure legend. A p-value less than 0.05 was considered statistically significant, with asterisks in the figures indicating the level of significance: ns for not significant, * for p < 0.05, ** for p < 0.01, *** for p < 0.001 and *** for p < 0.001.

## Data Availability

The datasets presented in this study can be found in online repositories. The RNA-seq data is accessible at Gene Expression Omnibus (GEO) platform, with accession number GSE284406. All R code and other scripts are available at: https://bitbucket.org/immunology-emc/bryostatin1_ctl/src/master/.
