# Supplementary material for: Bryostatin-1 enhances the proliferation and functionality of exhausted CD8+ T cells by upregulating MAP Kinase 11
Source: Front Immunol. 2025 Jan 14;15:1509874. doi: 10.3389/fimmu.2024.1509874 (PMC11772198; doi:10.3389/fimmu.2024.1509874)
Supplement: Supplementary file 1 [file DataSheet1.docx]

# Supplementary Figures


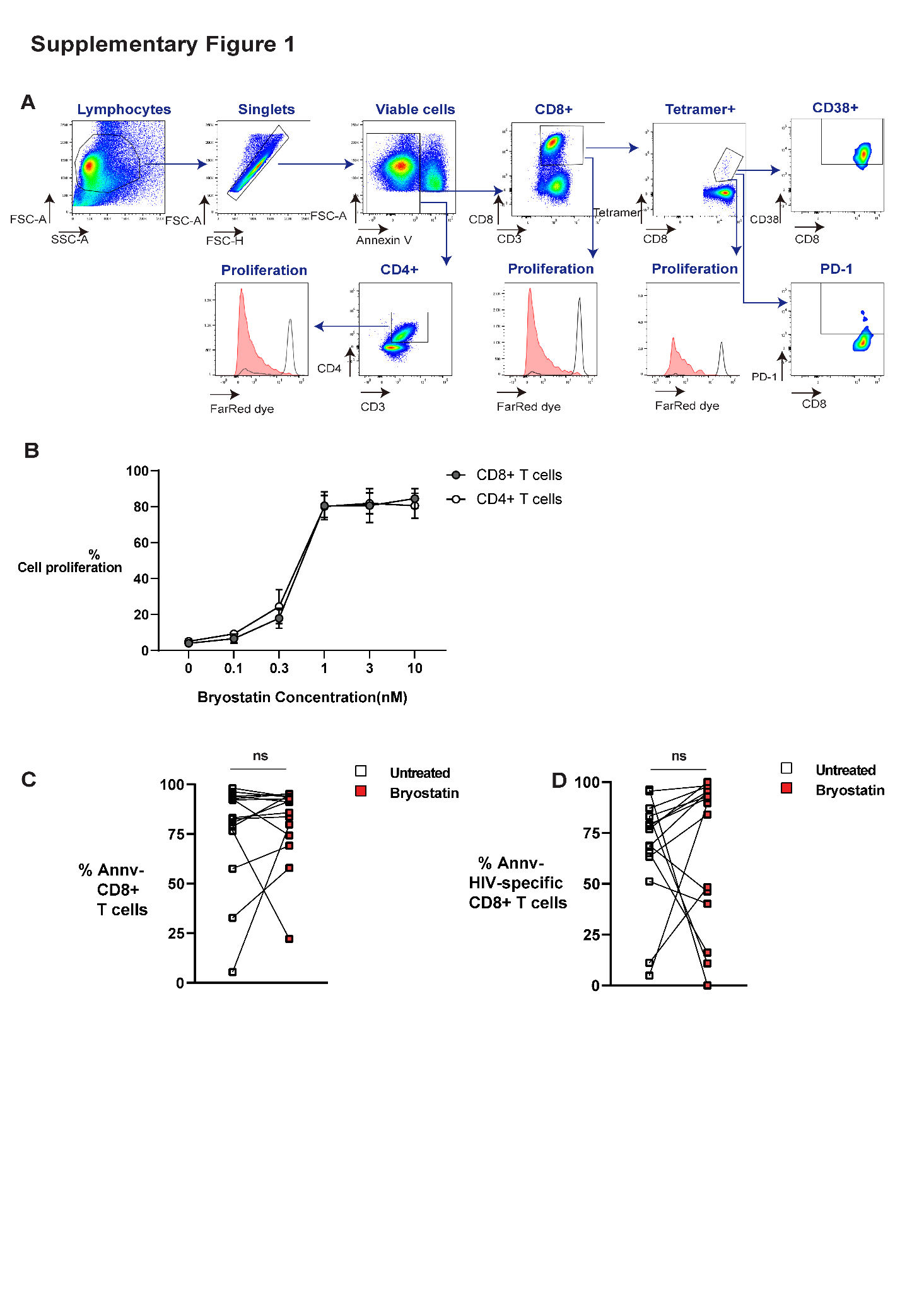


**Supplementary Figure 1.** **Low concentrations of bryostatin-1 proliferate T cells from PWH and bryostatin-1 did not significantly impact cell viability of CD8+ T cells and HIV-specific CD8+ T cells.** PBMCs from PWH were either untreated or treated with different concentration of bryostatin-1 from 0.1 nM to 10 nM in the presence of anti-CD28 costimulation for 6 days. Cells were labeled with Far Red proliferation dye on day 0 for tracking cell proliferation and cells were harvested for flow analysis on day 6. **(A)** Gating strategy for detecting different cell proportion, cell proliferation and surface markers in PBMCs from PWH. **(B)** Pooled data showing the proliferation of both CD8+ and CD4+ T cells treated with different concentrations of bryostatin-1. % Cell proliferation represents the percentage of CD4+ or CD8+ T cells which underwent at least one division with or without bryostatin-1 treatment during the six days culture. Vertical lines depict mean ±SE (n=4, 4 independent experiments). **(C)** Pooled data depicting the percentage of live total CD8+ T cells before and after 10 nM bryostatin-1 treatment. Live total CD8+ T cells were gated on CD3+ CD8+ Annexin V-. Each symbol represents one individual (n=15, 15 independent experiments). **(D)** The percentage of live (Annexin V negative) HIV-specific CD8+ T cells depicted before and after 10 nM bryostatin-1 treatment. Each symbol represents one individual (n=15, 15 independent experiments). Wilcoxon matched-pairs signed rank test was performed for statistical tests. ns for not significant.


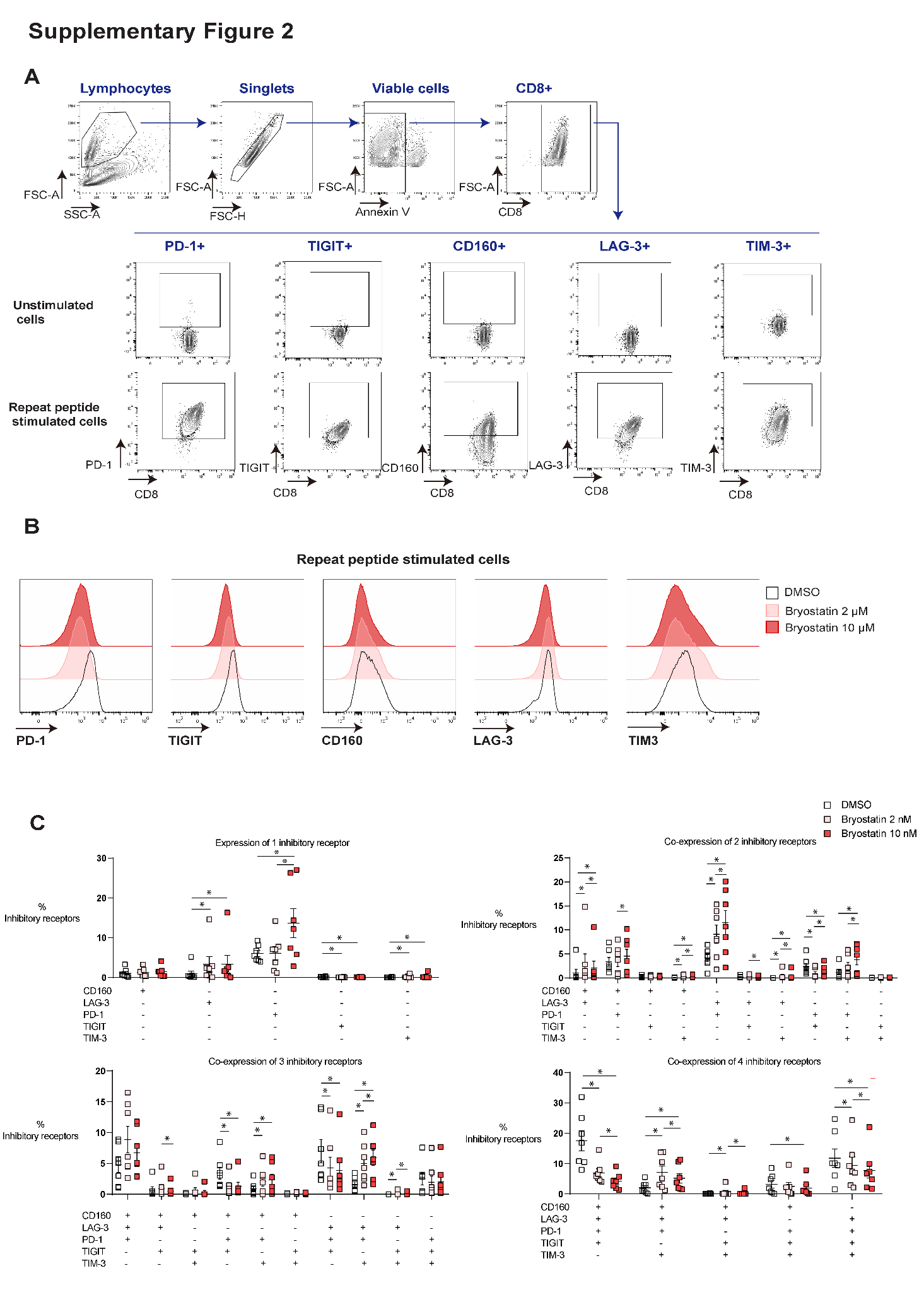


**Supplementary Figure 2. Bryostatin-1 decreases multiple inhibitory receptors in *in vitro* exhausted T cells. (A)** Gating strategy for detecting the expression of inhibitory receptors. Singlets were gated using FSC-A and FSC-H. Dead cells were excluded by Annexin V positive staining. Gating for inhibitory receptors was based on unstimulated cells. (B) Representative histograms depicting the expression of inhibitory receptors on DMSO, 2 nM bryostatin-1or 10 nM bryostatin-1 treated *in vitro* exhausted cells on day 8. (C) Figure depicting co-expression of inhibitory receptors, including CD160, LAG-3, PD-1, TIGIT and TIM-3, on DMSO, 2 nM bryostatin-1or 10 nM bryostatin-1 treated exhausted CD8+ T cells. Each square indicates an individual culture (n=7).


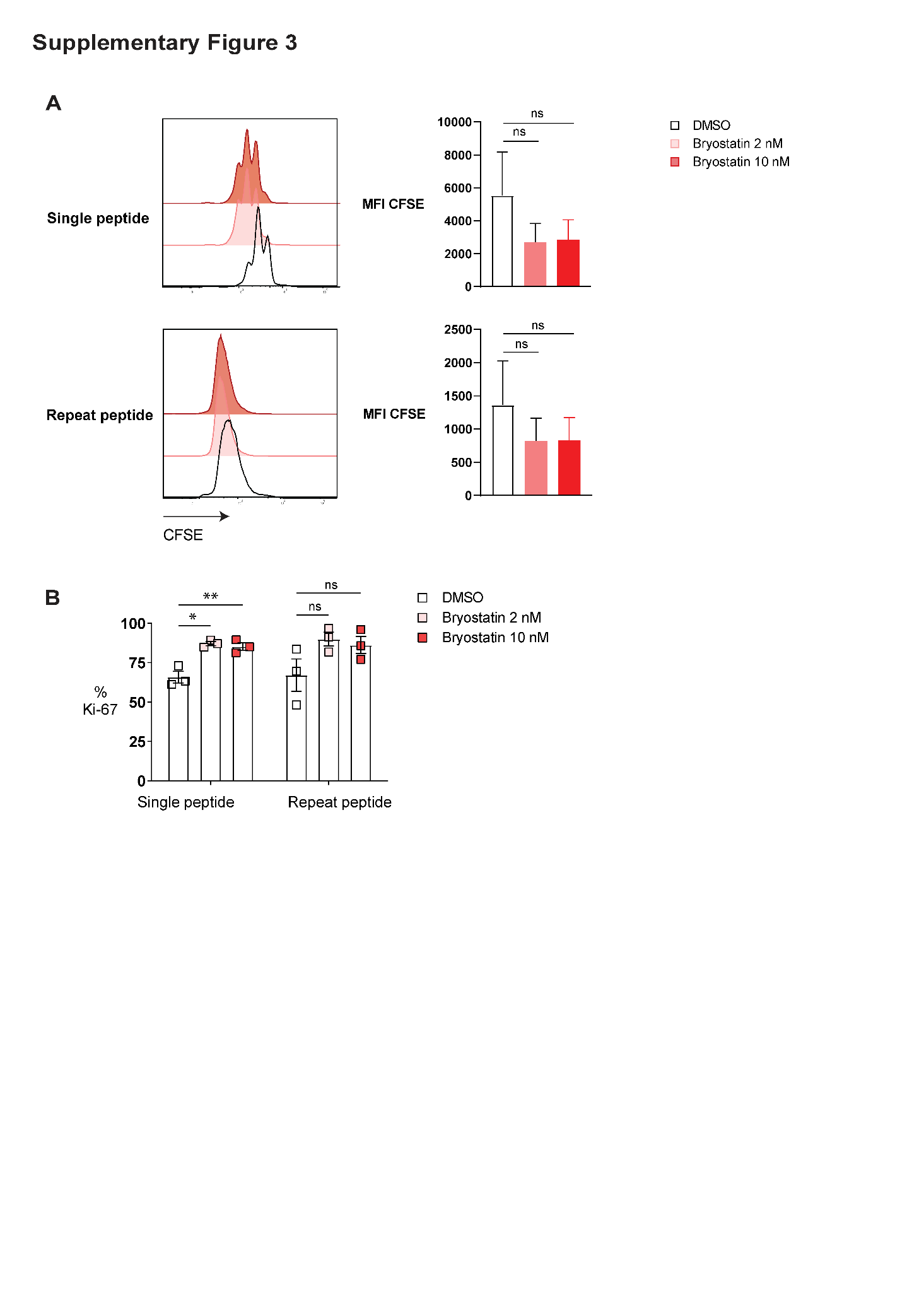


**Supplementary Figure 3. Bryostatin-1 enhances the proliferation of *in vitro* exhausted T cells.** T cell exhaustion was induced by daily OVA _(257-264)_ peptide stimulation for 5 days (repeat peptide stimulated condition). Single peptide stimulated cells did not lead to exhaustion and served as a control. From day 5, cells were either treated with 2 nM bryostatin-1, 10 nM bryostatin-1 or DMSO for additional 3 days. All cells were labelled with CFSE proliferation dye on day 5. **(A)** Representative histogram (left) and pooled data(right) depicting the proliferation of both single peptide stimulated CD8+ T cells and repeat peptide stimulated CD8+ T cells with/without bryostatin-1 treatment. Two independent experiments were performed. **(B)** The frequency of Ki-67+ CD8+ T cells shown for the different culture conditions. 3 independent experiments were performed. Statistical differences were determined by paired t test. ns for not significant, * for p < 0.05, ** for p < 0.01.


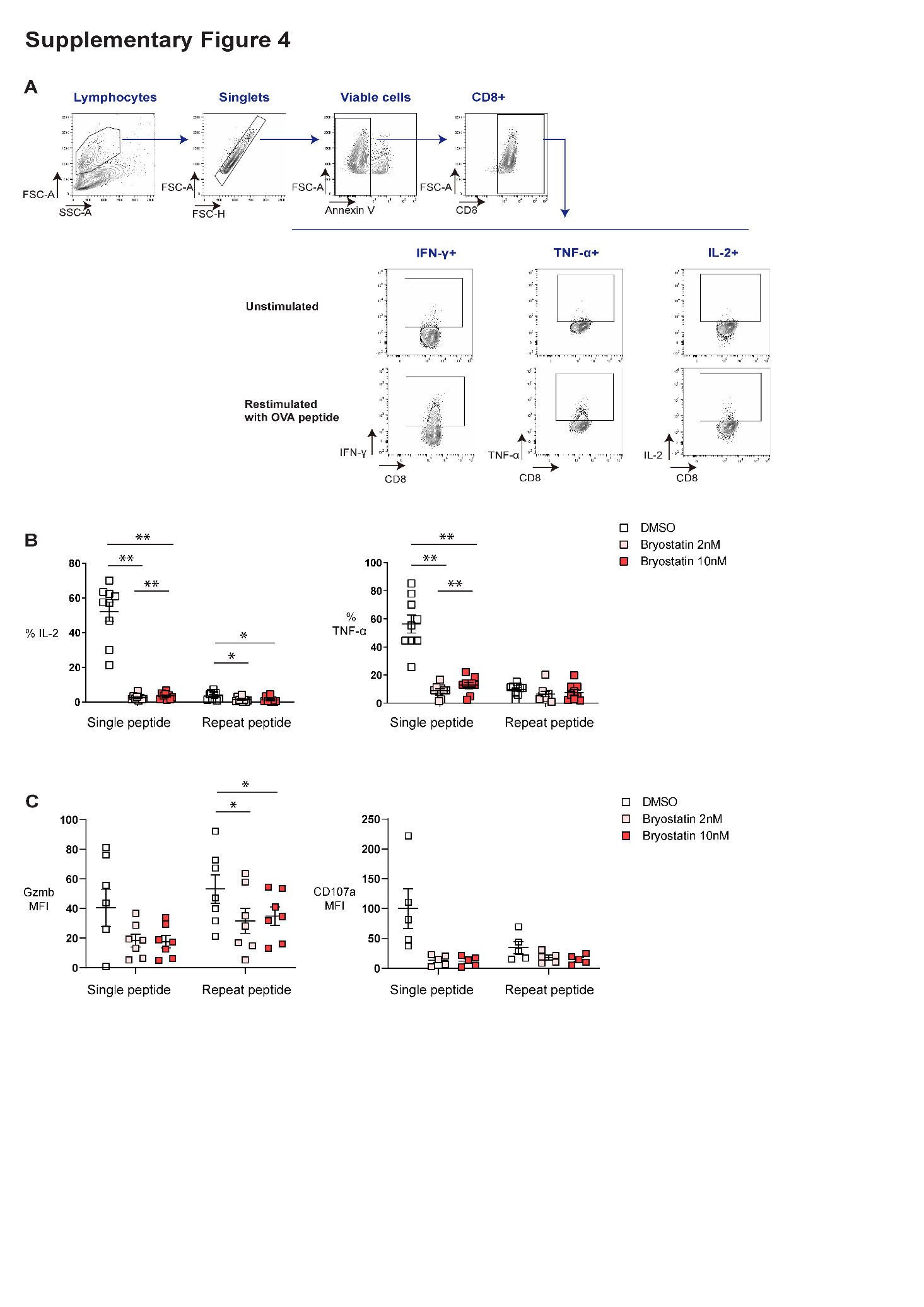


**Supplementary Figure 4. Bryostatin-1 does not restore IL-2 and TNF-α production of exhausted T cells. (A**) Gating strategy for expression of cytokines shown in exhausted T cells. Doublets were excluded using FSC-A and FSC-H, followed by Annexin V staining to identify live cells. Gating for cytokines was based on unstimulated cells. **(B)** Pooled data depicting the frequency IL-2 (left) and TNF-α (right) in cells treated either with DMSO or bryostatin-1. Each symbol represents one animal (n=7-10), 7 independent experiments were performed. **(C)** Pooled data depicting the granzyme B (Gzmb) MFI levels (left) and CD107a MFI (right) in cells treated either with DMSO or bryostatin-1. Each symbol represents one animal (n=5-10), 5-10 independent experiments were performed.


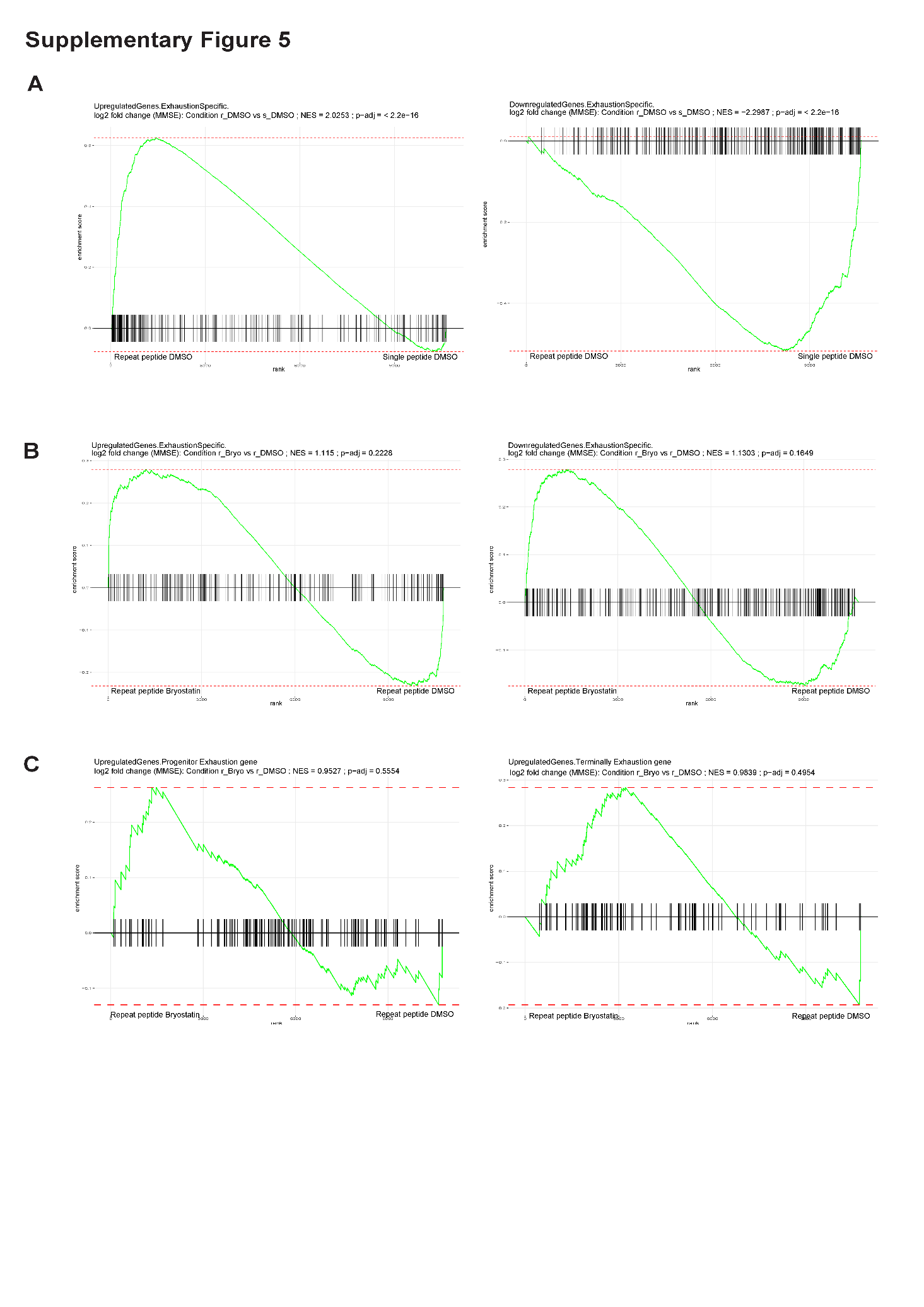


**Supplementary Figure 5. Bryostatin-1 does not reverse exhaustion profile of exhausted T cells.** (A) Enrichment of exhaustion gene signature derived from chronic LCMV-infection was analyzed in RNA-seq data from repeat peptide stimulated cells vs. single peptide stimulated cells. (B) Enrichment of exhaustion-specific signature was tested on differentially expressed genes from bryostatin-1-treated vs. DMSO-treated exhausted T cells by Gene Set Enrichment Analysis (GSEA). (C) Enrichment of progenitor exhausted T cell (left) and terminally exhausted T cell gen signature (right) derived from chronic LCMV-infection was tested in differentially expressed genes from bryostatin-1-treated vs DMSO-treated exhausted T cells by GSEA. NES, normalized enrichment score.


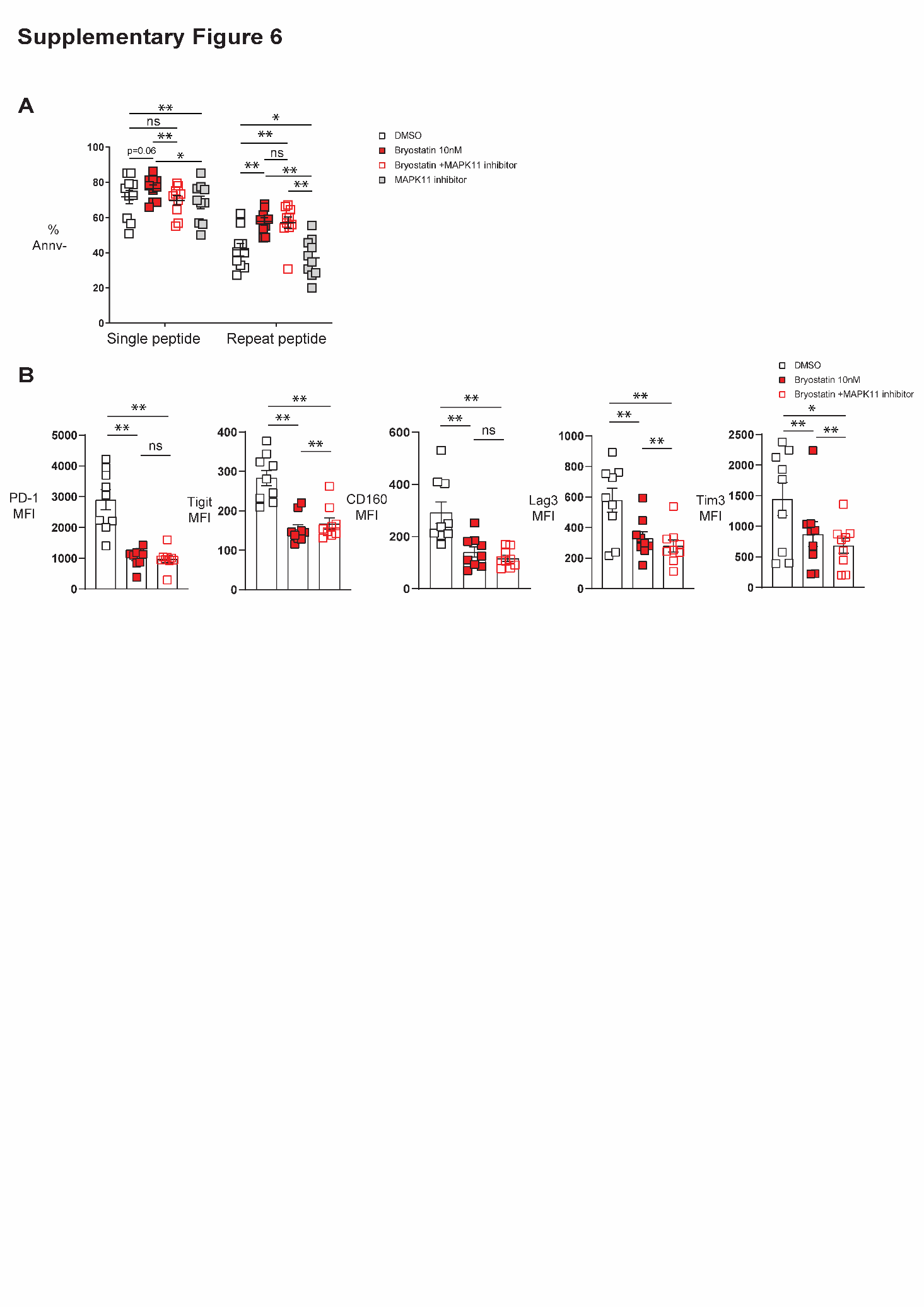


**Supplementary Figure 6. MAPK11 inhibition does not affect the cell viability of bryostatin-1 treated exhausted T cells and does not block the decreased inhibitory receptor expression by bryostatin-1 in these cells.** (A) Pooled data depicting the frequency of annexin V negative cells (Annv-) from the different culture conditions. (B) Pooled data showing the MFI of inhibitory receptor expression in bryostatin-1 treated exhausted T cells in the presence or absence of RWJ-67657. Each symbol represents one animal (n=9-10), 5 independent experiments were performed. Lines depict mean ±SE. Between groups, Wilcoxon matched-pairs signed rank test was performed for statistical analysis. ns for not significant, * for p < 0.05, ** for p < 0.01 and *** for p < 0.001.
